# Zinc versus Magnesium: Orthogonal Catalyst Reactivity in Selective Polymerizations of Epoxides, Bio‐derived Anhydrides and Carbon Dioxide

**DOI:** 10.1002/chem.201605690

**Published:** 2017-03-15

**Authors:** Prabhjot K. Saini, Giulia Fiorani, Robert T. Mathers, Charlotte K. Williams

**Affiliations:** ^1^ Department of ChemistryImperial College LondonLondonSW7 2AZUK; ^2^ Department of ChemistryUniversity of Oxford, Chemical Research Laboratory12 Mansfield RoadOxfordOX1 3TAUK; ^3^ Department of ChemistryThe Pennsylvania State UniversityNew KensingtonPennsylvania15068USA

**Keywords:** carbon dioxide, epoxides, ring-opening polymerization, terpenes, tricyclic anhydrides

## Abstract

Developing selective polymerizations from complex monomer mixtures is an important challenge. Here, dinuclear catalysts allow selective polymerization from mixtures of sterically hindered tricyclic anhydrides, carbon dioxide and epoxides to yield well‐controlled copoly(ester‐carbonates). Surprisingly, two very similar homogeneous catalysts differing only in the central metal, zinc versus magnesium, show very high but diametrically opposite monomer selectivity. The selectivity is attributed to different polymerization kinetics and to steric factors associated with the anhydrides.

Precise and selective polymerization methods have been long sought for the synthesis of complex architectures.^1^ The resulting polymers have potential to undergo controllable phase separations and self‐assembly^2^ and, in the case of oxygenated polymers, show tailored degradation rates and biocompatibilities.^3^, ^4^ While methods to synthesize low dispersity polymers are well established, there is still a need for methods to selectively enchain complex monomer mixtures. Since purification and separation are generally considered the most energy intensive and expensive facet of producing monomers, catalysts which selectively polymerize some monomers while ignoring others are important to improve sustainability and deliver selectivity.

Oxygenated block copolymer synthesis requires controlled polymerization methods, like ring‐opening polymerization (ROP) of cyclic esters/carbonates.3e, 4a, 5 The ring‐opening copolymerization (ROCOP) of epoxides/anhydrides and/or epoxides/carbon dioxide (CO_2_) is an interesting alternative route.^6^ ROCOP offers several benefits including: 1) a wide variety of polymerizable epoxides/anhydrides, many of which are commercially available; 2) a strong thermodynamic driving force enabling the polymerization of substituted and functionalized monomers; 3) incorporation of aromatic or rigid moieties into the polymer backbone to improve the overall thermal properties.6b Despite such attractive features, many opportunities exist to improve ROCOP catalysts, including the selective synthesis of stereoregular polyesters.^7^

Selective polymerizations starting from complex mixtures rely on catalyst selectivity. High degrees of selectivity are not so feasible using heterogeneous catalysts, such as the commonly employed double metal cyanides (DMCs), and generally much better polymerization control is possible using homogeneous catalysis.^8^ A key example is the zinc β‐diiminate catalysts for polymerizations of epoxides, anhydrides and CO_2_ reported by Coates and co‐workers. After complete consumption of the anhydride monomer (and formation of the polyester block) selective epoxide/CO_2_ copolymerization occurred with formation of the polycarbonate block.8o Since this initial report, several other homogeneous catalysts displaying similar selectivities have been developed, although in some cases there was some tapering of block structures.8d, 8s–8v, 8y, 8ab The observed reactivity is usually attributed to the anhydride reacting faster than CO_2_ with the metal alkoxide intermediate.8o, 9

Here, mixtures of sterically congested tricyclic anhydrides, cyclohexene oxide (CHO) and CO_2_ are selectively polymerized using a magnesium (**1**)^10^ or zinc (**2**)^11^ catalyst (Scheme 1). Both catalysts are active towards CHO/CO_2_ ROCOP, operating efficiently at 1 bar CO_2_ pressure, as well as for CHO/phthalic anhydride (PA) ROCOP.8aa, 10, 11 The tricyclic bio‐derived anhydride BCA1 was readily synthesized from α‐phellandrene (a mono‐terpene extracted from *Eucalyptus radiate*) and maleic anhydride (MA) via an atom efficient Diels–Alder reaction.^12^ Structurally similar tricyclic anhydrides (BCA2, BCA3 and CA) were deliberately selected so as to increase both the renewable content and the thermal resistance of the polymers by incorporating rigid repeating units. Recently, related bio‐derived tricyclic anhydrides were applied, with epoxides, to prepare polyesters with increased thermal stabilities.8q, 8r, 13

**Scheme 1:**
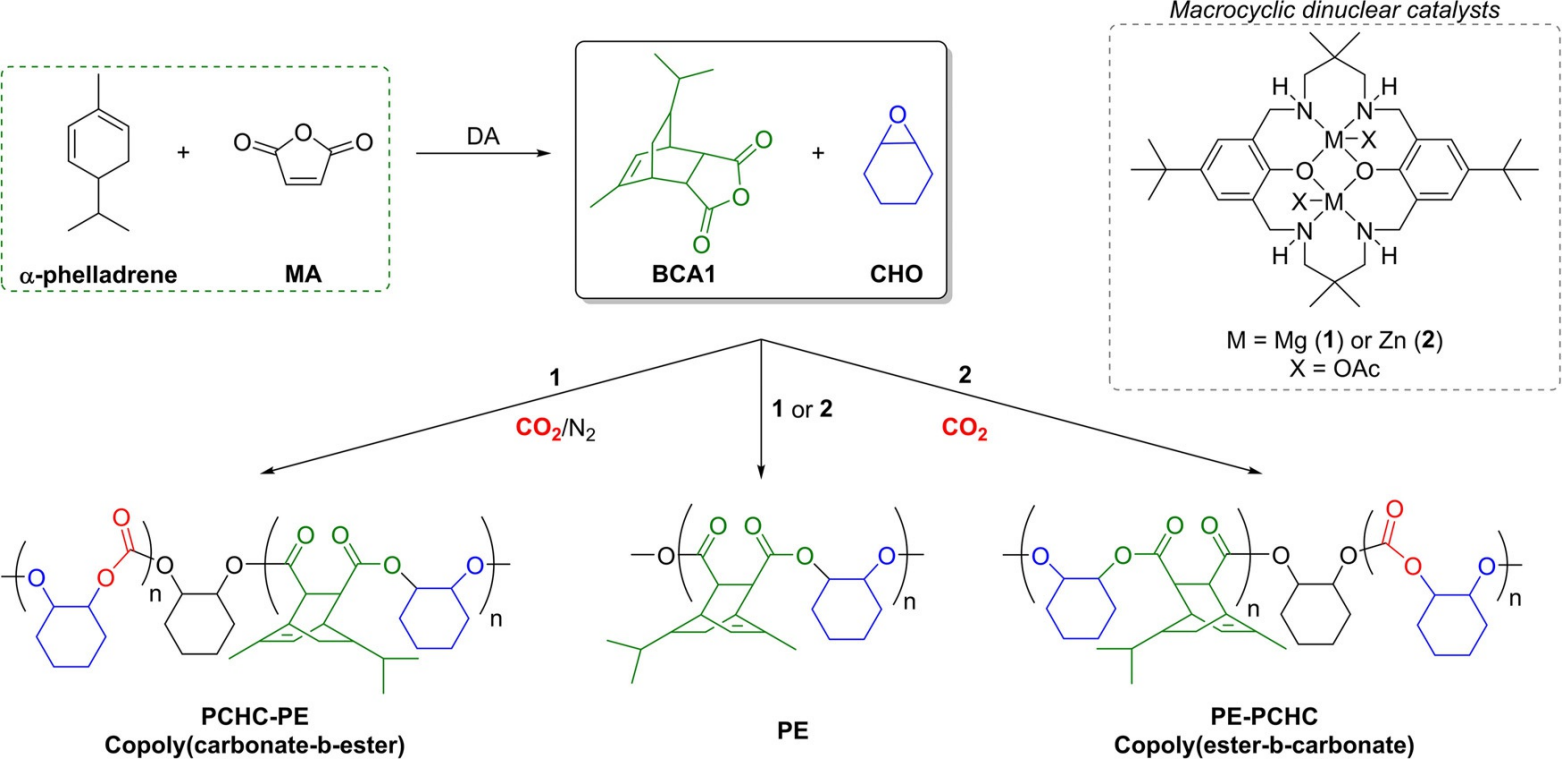
The bio‐derived anhydride, BCA1, and polyesters and block copolymers produced using selective catalysis. The structures of catalysts **1** and **2** are illustrated in the box.^10^, ^11^

Initially, the polymerization of CHO and BCA1 was investigated: both catalysts showed good activities and yielded polymer chains with >99 % ester linkages (entries 1 and 2, Table 1 and Figures S2 and S5). The polymer molecular weights show clear bimodal distributions with the higher distribution being approximately double the molecular weight of the lower (Table 1 and Figures S3 and S4).

**Table 1:** Polymerizations of anhydride (BCA1), epoxide (CHO) and CO_2_ initiated by **1** and **2**.

Cat.^[a]^	*t* [h]	% Ester linkages (% carbonate linkages in the block copolymers)^[b]^	*M_n_* (*Ð*)^[c]^
1*	1.3	>99 %	7750 (1.14)
			4180 (1.42)
			
2*	22	>99 %	7460 (1.08)
			4320 (1.24)
			
**1**	1.16	0 (100 % carbonate)	7810 (1.15)
			4410 (1.46)
		
	3.16	34 % (66 % carbonate)	11 800 (1.13)
			6440 (1.34)
			
**2**	22	100 % (0 % polycarbonate)	3040 (1.23)
			4860 (1.07)
	27.8	35 % (65 % polycarbonate)	4950 (1.31)
			7830 (1.10)
			
**1** ^[d]^	6	0 (100 % carbonate)	3020 (1.09)
	22.8	49 % (51 % carbonate)	6340 (1.11)

Polymerization conditions: catalyst/BCA1/CHO/CO_2_=1:100:1000:1 atm, 100 °C. All experiments were allowed to reach >98 % anhydride (BCA1) conversion (by NMR). *No CO_2_ used in reaction. [a] TOF=[# moles monomer converted/# moles catalyst]/time [h]. [b] Determined by integrating the normalized resonances for ester linkages (4.78–4.50 ppm) against ether linkages (3.50–3.30 ppm) or carbonate linkages (4.80–4.40 ppm). [c] Determined by SEC in THF calibrated using polystyrene. Note that the MW distributions are fit using Gaussian distributions to obtain the Đ values (Figures S18–S25). [d] Using cyclohexanediol (CHD) as the chain transfer agent. Polymerization conditions: catalyst/CHD/BCA1/CHO/CO_2_=1:20:400:1500:1 atm, 100 °C.

Furthermore, each of the polymer molecular weights (MW) are lower than the calculated theoretical values (based on equiv catalyst vs. monomer conversions). Both observations are fully consistent with the published literature in the field of cyclohexene oxide/anhydride copolymerizations and can be rationalized by chain growth initiated from both the catalyst and 1,2‐cyclohexanediol (CHD). The diol species is proposed to form by a side‐reaction between CHO and residual water.8d–8ad, 10 Indeed, a recent spectroscopic study using Cr‐salen catalysts revealed that hydrolysis occurs prior to any polymerization.^14^ In light of this, it is proposed that initiation from the acetate groups gives rise to the lower MW distribution corresponding to α‐acetate‐ω‐hydroxyl‐polyester, whilst the higher MW distribution corresponds to telechelic chains of α,ω‐di‐hydroxyl‐polyesters as depicted in Figure S12. Indeed, both the bimodal distribution and the two different end‐groups were also confirmed by MALDI‐ToF analysis (Figure S27).8aa

Considering the catalysts activities, the macrocyclic dinuclear Mg catalyst **1** is about 17 times faster than **2**, which is in agreement with previous reports on CHO/CO_2_ and CHO/phthalic anhydride copolymerizations.8aa, 10, 11 The absolute TOF value for **1** (77 h^−1^) is similar to other catalysts for epoxide/anhydride copolymerizations, albeit tested using different anhydrides.6b Both catalysts show excellent selectivity for ester linkages (>99 %) similar to the most selective and active Al‐salen catalysts reported so far.8q, 8r, 13 Notably, **1** does not require any co‐catalyst as co‐catalysts are known to cause side‐reactions.6b, 8h Next, both catalysts were tested using mixtures of BCA1, CHO and CO_2_. Both catalysts yielded block copolymers with high selectivity but with exactly opposite monomer enchainment (Scheme 1, bottom). The Zn based catalyst **2** firstly underwent epoxide/anhydride polymerization until all the anhydride was consumed, after which alternating epoxide/CO_2_ polymerization occurred. The polymerization was monitored using in situ ATR‐IR spectroscopy (Figure 1, bottom), which showed that the anhydride was consumed first, as evidenced by a sharp decrease in the absorbance at 1800–1770 cm^−1^. Once consumed, polycarbonate formation occurred, as shown by an increase in the absorption at 1239–1176 cm^−1^. The IR assignments were verified by independent analysis of control polymers. Furthermore, aliquots were regularly withdrawn and analysed using ^1^H NMR spectroscopy (Figure S5). The NMR spectra confirmed polyester formation, as evidenced by a decrease in intensity of the BCA1 monomer resonance at 5.77 ppm, and by the appearance of typical polyester resonance signals at 5.85–5.45 ppm. Once the anhydride was consumed, polycarbonate formation occurred as shown by the appearance of new signals at 4.82–4.46 ppm. DOSY analysis supported block copolymer formation (Figure S6). The observed monomer selectivity was similar to previously reported homogeneous catalytic systems.6b, 8o

**Figure 1:**
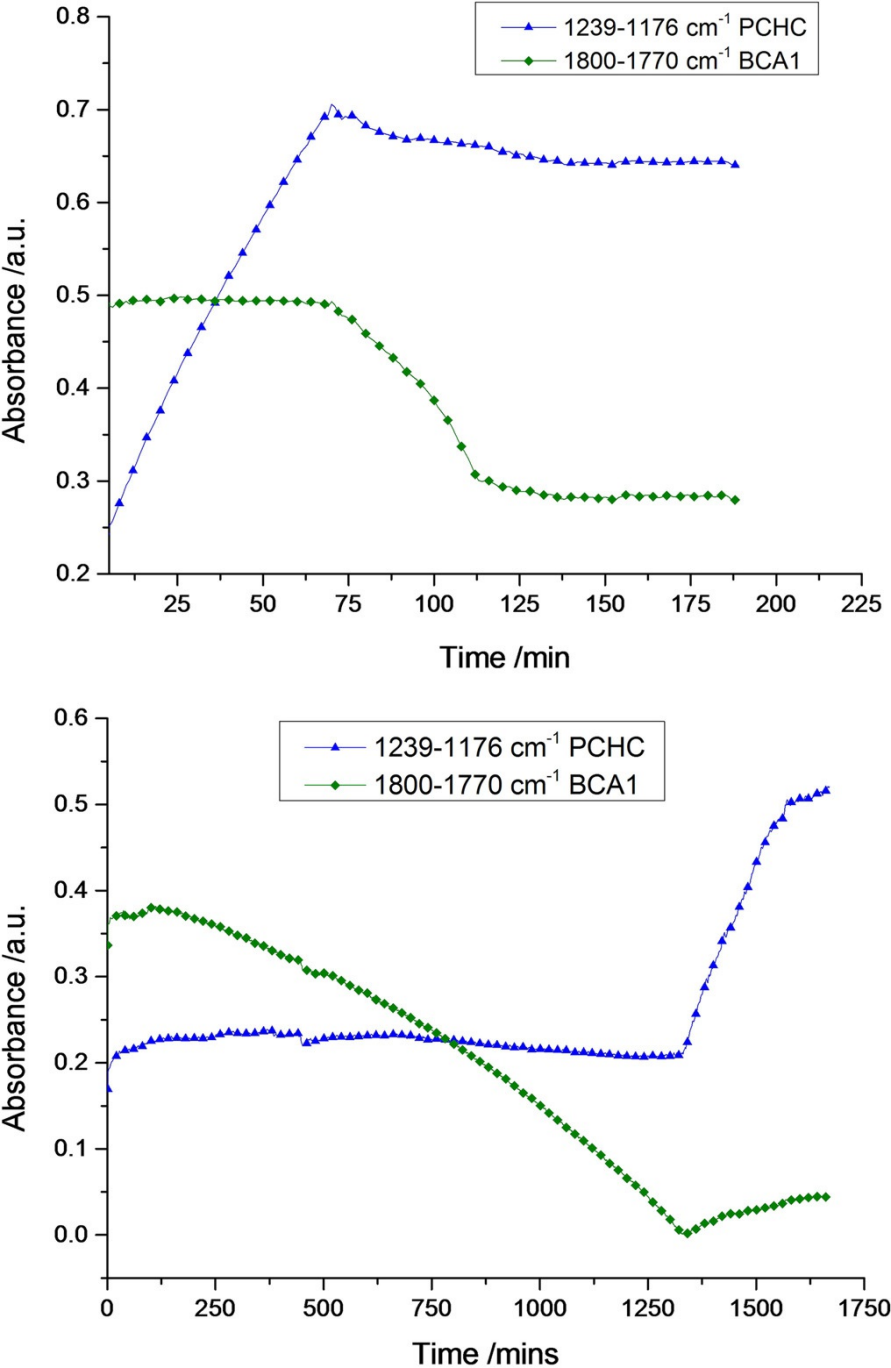
In situ ATR‐IR monitoring of polymerization reactions using: top) catalyst **1**, and bottom) catalyst **2**. Polymerization conditions: catalyst/BCA1/CHO/CO_2_=1:100:1000:1 atm, 100 °C, in neat CHO.

In contrast, Mg‐based catalyst **1** showed exactly the opposite reactivity. Surprisingly, the polycarbonate block formed first, as shown by the IR absorption spectrum (Figure 1, top): while the absorbance of BCA1 (1800–1770 cm^−1^) remained unchanged polycarbonate absorption (1239–1176 cm^−1^) increased. ^1^H NMR analysis showed that the intensity of BCA1 resonance at 5.77 ppm did not change whilst those of the polycarbonate (4.82–4.46 ppm) increased (Figure S7). After 6 h, the excess CO_2_ was removed by five rapid vacuum/N_2_ cycles, and epoxide/anhydride copolymerization immediately occurred. Without CO_2_ being present, the resonance at 1800–1700 cm^−1^ decreased rapidly (Figure 1, top). Polymerizations at twice the concentration of anhydride were conducted but the carbonate block was still formed first (Figure S8).

The selectivity of **1** is very unusual and has not been observed with any other catalyst.6b To confirm block copolymer formation, the molecular weights (MW) of aliquots were analysed. The MW increased before and after block formation, however, the distributions were bimodal (Figures S9 and S10). DOSY NMR analysis indicated block copolymer formation and a single diffusion coefficient was observed (Figure S11).8ad, 15 The observed bimodality in the molecular weight distributions of the polyesters is expected to result in the formation of a mixture of AB and ABA type block copolymers when poly(ester carbonates) are produced. To selectively form ABA triblocks, polymerizations were conducted using excess CHD as the chain transfer agent (20 equiv CHD vs. **1**). Under such conditions, all aliquots show monomodal MW distributions, with narrow dispersities (Figure S13). DOSY NMR suggests both polymers are attached which would be in line with a block copolymer structure (Figure S15).

Kinetic investigations of epoxide/anhydride polymerizations suggested that catalyst **1** obeyed a first order rate dependence on anhydride concentration. Semi‐logarithmic conversion versus time data obeyed a linear fit after a short induction period (Figure 2, top). It should be noted that the induction period of >20 min corresponds to the time required for both thermal equilibration and complete dissolution of the anhydride. On the other hand, the kinetic data obtained using catalyst **2** could be fit with a zero order rate dependence on anhydride concentration, that is, a linear fit was obtained to conversion versus time data (Figure 2, bottom). The first order dependence is very unusual as most of the other known catalysts show a zero order dependence on anhydride concentration.8d, 8s–8v, 8y, 8aa Furthermore, using either **1** or **2** in CHO/PA ROCOP showed zero order dependence under all conditions, thereby highlighting the influence of the chemistry of the anhydride (Table 2).8aa

**Figure 2:**
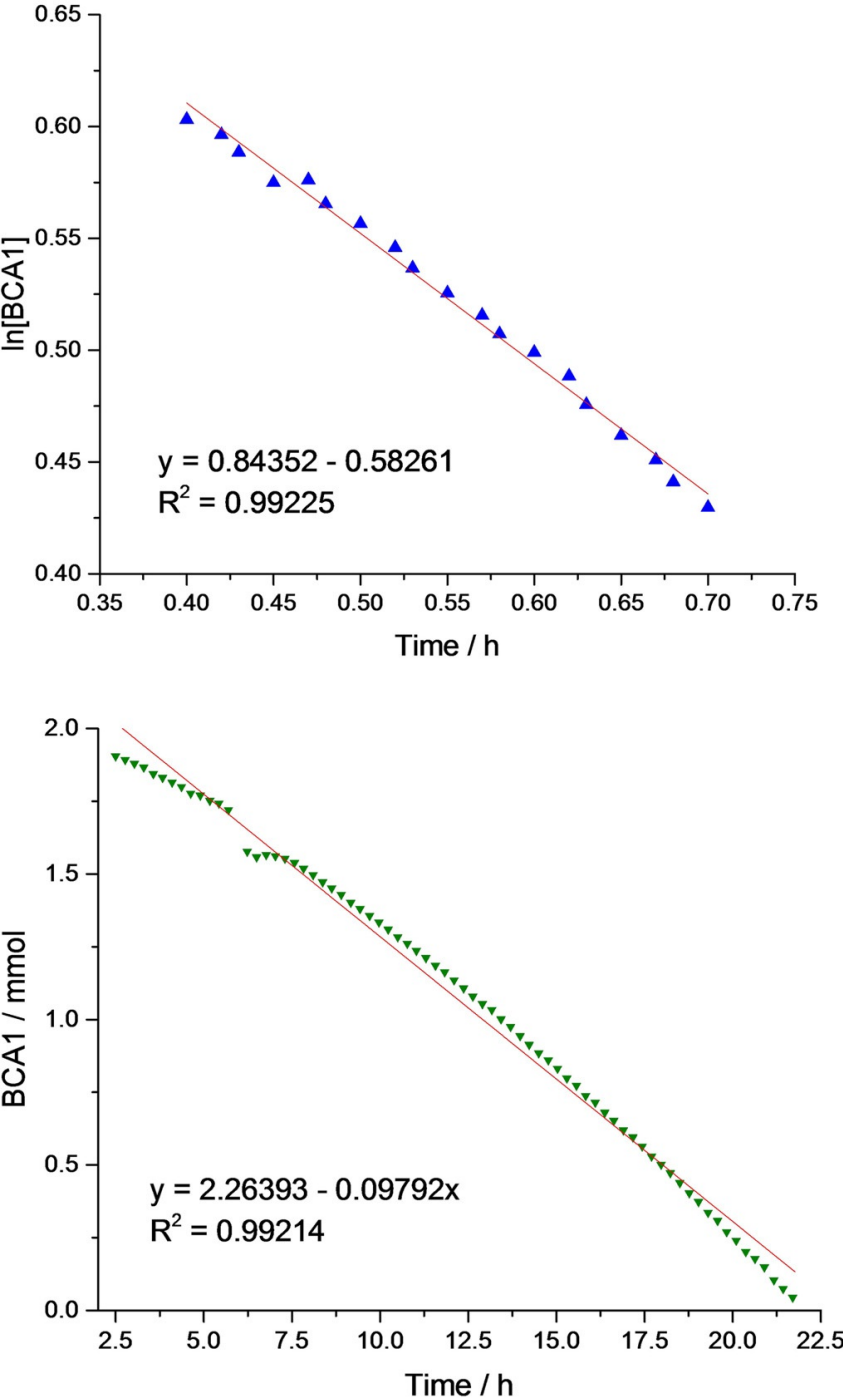
Top) Plot of ln([BCA1]) versus time for catalyst **1** (first order monomer dependence), and bottom) plot of [BCA1] versus time for catalyst **2** (zeroth order monomer). Polymerization conditions: [catalyst]/[BCA1]/[CHO]=1:100:1000, 100 °C, in neat CHO.

**Table 2:** The ROCOP of BCA1/CHO initiated by **1** and **2**.

Catalyst	Polymer	*M_n_* (*Ð*)^[a]^	% Ester^[b]^	*T*_g_^[c]^
**2**	PE	5930 (1.08), 2530 (1.09)	100	118
**1**	PE	6730 (1.06), 2870 (1.09)	100	117
**1**	PE	14 760 (1.15), 4350 (1.11)	100	126
**1**	PC‐b‐PE	6230 (1.16)	60	113
**2**	PE‐b‐PC	10460 (1.06), 4280 (1.09)	30	95
[Cr]8q	PE8q	21900 (1.26)	100	86

[a] Determined by SEC, using polystyrene calibration. [b] Determined by integrating the normalized resonances for ester linkages (4.78–4.50 ppm) in the ^1^H NMR spectrum. [c] Determined by DSC, from the second and third cycles.

A possible model which could rationalize the kinetic data is illustrated in Figure 3. According to the hypothesis, the two polymerizations cycles are linked by a common metal–alkoxide intermediate which can react either with a) with an anhydride to form ester linkages (*k*_1_) or b) with CO_2_ to carbonate linkages (*k*_1′_). It would be useful to directly determine the rates of the insertion reactions and such aim is part of our long‐term research endeavours. In this context, it is relevant to note that direct rate determination is not straightforward and recent work on the rate of CO_2_ insertion into Zn−H bonds highlighted the complexities of such measurements, with the apparent rate being fast but ultimately diffusion limited.^16^ In place of direct rate measurements, the reaction orders in monomer concentrations are relevant to rationalize the opposite selectivities observed using the two catalysts. Both catalysts are already investigated for carbon dioxide/epoxide copolymerizations, with the di‐zinc species showing a zero order dependence on carbon dioxide pressure over the range 1–40 bar^17^ and the magnesium catalyst showing TOF values which were not substantially affected by pressure. Thus, it is proposed that both catalysts show rapid insertion of carbon dioxide during CHO/CO_2_ copolymerizations. Considering the dependence on anhydride concentrations, catalyst **2** showed an apparent zeroth dependence on anhydride concentration. During terpolymerization experiments the anhydride was consumed prior to CO_2_, indicating that anhydride insertion occurs faster than CO_2_ insertion from the common alkoxide intermediate (i.e., *k*_1_ > *k*_1′_, Figure 3). Such a rationale is in agreement with the mechanism proposed by Coates and co‐workers in their seminal studies of block selectivity in terpolymerizations using phthalic anhydride.8o In contrast, when using catalyst **1** a first‐order dependence on anhydride concentration appeared a more suitable fit to the data. Furthermore, the terpolymerization data showed that CO_2_ was inserted prior to anhydride consumption. Indeed, anhydride consumption only occurred after CO_2_ removal from the reaction solution. These findings as a whole may indicate that the rate of CO_2_ insertion from the common alkoxide intermediate is greater than that of anhydride insertion (i.e., *k*_1′_>*k*_1_, Figure 3). Given that **1** and **2** have the same ligand and only differ by the choice of metal, the opposite selectivities towards monomers was unexpected.^10^, ^11^, ^18^ Furthermore, the anhydride rigid and hindered structure also seems to be important in determining process selectivity. To support this observation, other tricyclic anhydrides featuring different steric shielding effects were tested using Mg‐catalyst **1** (Figure 3). When tested with mixtures of anhydride, CO_2_ and epoxide, catalyst **1** showed the unusual carbonate selectivity prior to anhydride enchainment with all the cyclic anhydrides (Figure S16). In contrast, the same catalyst exposed to *cis*‐1,2,3,6‐tetrahydro phthalic anhydride (THPA), CO_2_ and CHO showed anhydride consumption prior to CO_2_ enchainment (Figure S17). It is also relevant to note that terpolymerizations using phthalic anhydride, CO_2_ and CHO were already investigated using catalyst **1** and showed anhydride enchainment prior to CO_2_ polymerization.8aa Thus, the rigidity and steric hindrance of the tricyclic anhydrides are implicated in the unusual selectivity observed using catalyst **1**.

**Figure 3:**
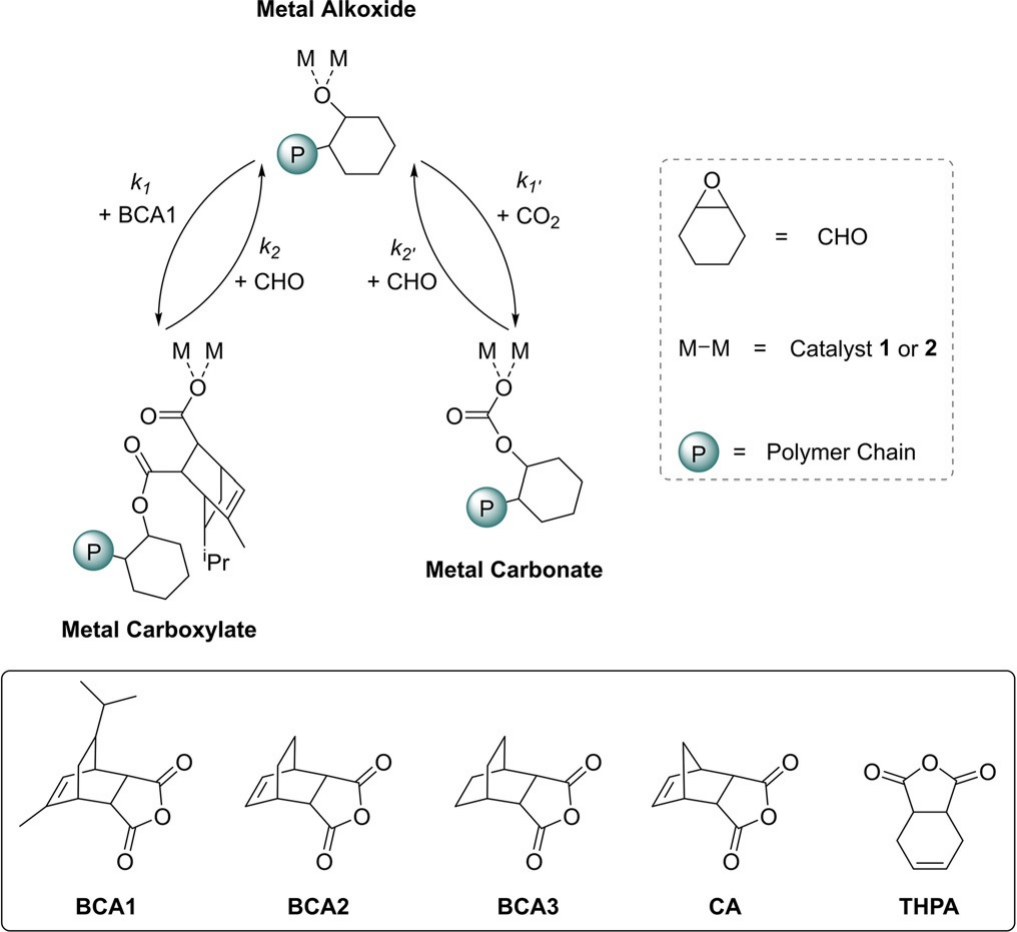
Structures of the key intermediates in the catalytic cycles (top) and bicyclic anhydrides used in this study (bottom).

The use of sterically encumbered bio‐derived tricyclic anhydrides allows incorporation of two rigid groups (epoxide and anhydride) into the polymer backbone which is expected to increase the polymer *T*_g_ values. Recent work from Coates, Kleij and co‐workers has also allowed preparation of various polyesters and carbonates using closely related tricyclic anhydrides. In that study, the authors observed enhanced *T*_g_ values for the materials.^13^ It is particularly important to increase the thermal resistance as many bio‐sourced polyesters suffer from lower *T*_g_ values: for example, polylactide has a *T*_g_<60 °C which limits some applications in hot/humid climates. DSC analyses of all the newly synthesised block copolymers showed high *T*_g_ values which could be further controlled by the carbonate/ester block ratio. In particular, materials with greater amounts of carbonate blocks (>70 %) showed slightly lower *T*_g_ values whilst those containing greater proportions of ester blocks (>60 %) showed *T*_g_ values up to 113 °C. The *T*_g_ of the polyesters are >30 °C higher than related bio‐derived anhydrides copolymerized with propylene oxide.8q, 13

In conclusion, dinuclear Zn and Mg based catalysts coordinated by the same ancillary ligand, showed unprecedented opposite selectivities in polymerizations of epoxide/bicyclic anhydrides and CO_2_ to yield block copoly(ester‐carbonates). When using the Zn based catalyst, epoxide/anhydride enchainment occurs first and only once anhydride is consumed, does epoxide/CO_2_ polymerization occur. In contrast, with the Mg based catalyst epoxide/CO_2_ enchainment occurs first and epoxide/anhydride polymerization follows only after CO_2_ removal. The observed selectivity is supported by different anhydride consumption kinetic profiles, observing an anhydride monomer reaction order dependence on the metal catalyst selected. Such selectivity has implications for controlling the position of blocks in more complex enchainment patterns and will form the basis of future investigations into controlled composition materials.

## Conflict of interest

The authors declare no conflict of interest.

## Supporting information

As a service to our authors and readers, this journal provides supporting information supplied by the authors. Such materials are peer reviewed and may be re‐organized for online delivery, but are not copy‐edited or typeset. Technical support issues arising from supporting information (other than missing files) should be addressed to the authors.

Supplementary
