# Supplementary material for: Zinc versus Magnesium: Orthogonal Catalyst Reactivity in Selective Polymerizations of Epoxides, Bio‐derived Anhydrides and Carbon Dioxide
Source: Chemistry. 2017 Mar 15;23(18):4260–5. doi: 10.1002/chem.201605690 (PMC5434931; doi:10.1002/chem.201605690)
Supplement: Supplementary file 1 — Supplementary [file CHEM-23-4260-s001.pdf]

CHEMISTRY
A European Journal

Supporting Information

Zinc versus Magnesium: Orthogonal Catalyst Reactivity in Selective Polymerizations of Epoxides, Bio-derived Anhydrides and Carbon Dioxide

Prabhjot K. Saini,[a] Giulia Fiorani,[b] Robert T. Mathers,*[c] and Charlotte K. Williams*[b]

chem_201605690_sm_miscellaneous_information.pdf

Table of Contents

Experimental Section……p. S2

Figure S1……p. S4

Figure S2……p. S5

Figure S3……p. S5

Figure S4……p. S6

Figure S5……p. S6

Figure S6……p. S7

Figure S7……p. S8

Figure S8……p. S9

Figure S9……p. S9

Figure S10……p. S10

Figure S11……p. S10

Figure S12……p. S11

Figure S13……p. S11

Figure S14……p. S12

Figure S15……p. S12

Figure S16……p. S13

Figure S17……p. S14

Figure S18……p. S15

Table S1……p. S16

Figure S19……p. S17

Figure S20……p. S18

Figure S21……p. S19

Figure S22……p. S20

Figure S23……p. S21

Figure S24……p. S22

Figure S25……p. S23

Figure S26……p. S24

References……p. S25

Experimental Section

Materials and Methods

All experiments were carried out under N_2 using standard Schlenk techniques or in a Mbraun Unilab glovebox. The catalysts [$LMg_2(OAc)_2$] (**1**) and [$LZn_2(OAc)_2$] (**2**) were synthesized employing previously published methods.[1] All solvents and reagents were purchased from commercial sources (Sigma Aldrich and Alfa Aesar) and used as received, unless stated otherwise. Cyclohexene oxide (CHO) was dried over CaH_2, fractionally distilled and stored under a nitrogen atmosphere. Research grade CO_2 (99.99995%) for copolymerization reactions was purchased from BOC (Linde Gas). BCA1-3 were synthesised according to published procedures.[2] Carbic anhydride and cis-1,2,3,6-tetrahydro phthalic anhydride (THPA) were purified by dissolving in benzene, filtering off the insoluble impurities, removing the solvent and drying the monomers under vacuum on a Schlenk line at 30 °C for 48 h.

^{1}H NMR spectra were measured in $CDCl_3$ on a Bruker AV-400 spectrometer. *In situ* ATR-FTIR measurements were performed on a Mettler-Toledo ReactIR 4000 spectrometer equipped with a MCT detector and a silver halide DiComp probe for *in situ* ATR-FTIR measurements. Size exclusion chromatography (SEC) data was collected using a Shimadzu LC-20AD, with two Mixed Bed PSS SDV linear S columns with THF as the eluent, at a flow rate of 1 mL min^{-1} at 30°C and narrow M_n polystyrene standards for calibration. The data are analysed using the instrument software (*Shimadzu LabSolutions Main – GPC Postrun)* which was used to determine M_n, M_w and Đ values for the samples. It is relevant to note that where bimodal distributions are observed, the software automatically deconvolutes and models the distributions. Here, we also include the raw data fit with Guassian distributions – the values obtained for M_n and Đ are within error of those obtained directly from the instrument software. MALDI-ToF spectrometry measurements were performed on Waters/Micromass MALDI micro MX spectrometer. The samples were dissolved in THF and mixed with an appropriate matrix (dithranol).

General Copolymerization Procedure (Table 1)

The anhydride (1.98 mmol, 100 eq.) was added to a two neck Schlenk tube in the glovebox and the probe of the ATR-IR spectrometer was placed in the vessel. The system was dried for 16 h under vacuum at 30 °C. A solution of cyclohexene oxide (2 mL, 19.8 mmol, 1000 eq.) and catalyst (19.8 µmol) was added, under N_2, to the Schlenk tube. The vessel was heated to 100 °C, with constant stirring, and allowed to react until complete conversion of the anhydride (1.3 h for **1** or 24 h for **2**). A sample of the reaction mixture was analysed by ^{1}H NMR spectroscopy and size exclusion

chromatography. The volatile components were removed, *in vacuo,* to yield the product as a white powder. The crude polymer was purified by dissolving in THF and precipitating from pentane. A THF solution of the polymer was also passed through a silica plug in order to remove any catalyst residues.

General Terpolymerisation Procedure

The anhydride (1.98 mmol, 100 eq.) was added to a two neck Schlenk tube in the glovebox and the probe of the ATR-IR spectrometer was placed in the vessel under N_2. The system was dried for 16 h under vacuum at 30 °C. A solution of cyclohexene oxide (2 mL, 19.8 mmol, 1000 eq.) and catalyst (19.8 μmol) was added, under 1 atm pressure of CO_2, to the Schlenk tube. The vessel was heated to 100 °C, with constant stirring, for 27 h when using catalyst **2**. For catalyst **1**, after ≈ 3 h the reaction mixture was degassed and refilled with N_2. The reaction was left under N_2, at 100 °C and with continuous stirring until the anhydride had fully consumed (≈2 h). A sample of the crude reaction mixture was analysed by ^{1}H NMR spectroscopy and size exclusion chromatography. Any unreacted monomers were removed, *in vacuo,* to yield the product as an off white powder. The polymer was purified by precipitation from THF solution using pentane and passing a THF solution of the polymer through a silica plug.

ABA triblock terpolymerization procedure

The anhydride (1.98 mmol, 100 eq.) was added to a two neck Schlenk tube in the glovebox and the probe of the ATR-IR spectrometer was placed in the vessel under N_2. The system was dried for 16 h under vacuum at 30 °C. A solution of cyclohexene oxide (2 mL, 19.8 mmol, 1000 eq.), 1,2-cyclohexenediol (46 mg, 0.6 mmol, 20 eq.) and catalyst **1** (19.8 μmol) was added, under 1 atm pressure of CO_2, to the Schlenk tube. The vessel was heated to 100 °C, with constant stirring, for 3 h, after which the reaction mixture was degassed and refilled with N_2. The reaction was left under N_2, at 100 °C and with continuous stirring until the anhydride had fully consumed (≈2 h). A sample of the crude reaction mixture was analysed by ^{1}H NMR spectroscopy and size exclusion chromatography. Any unreacted monomers were removed, *in vacuo,* to yield the product as an off white powder. The polymer was purified by precipitation from THF solution using pentane and passing a THF solution of the polymer through a silica plug.

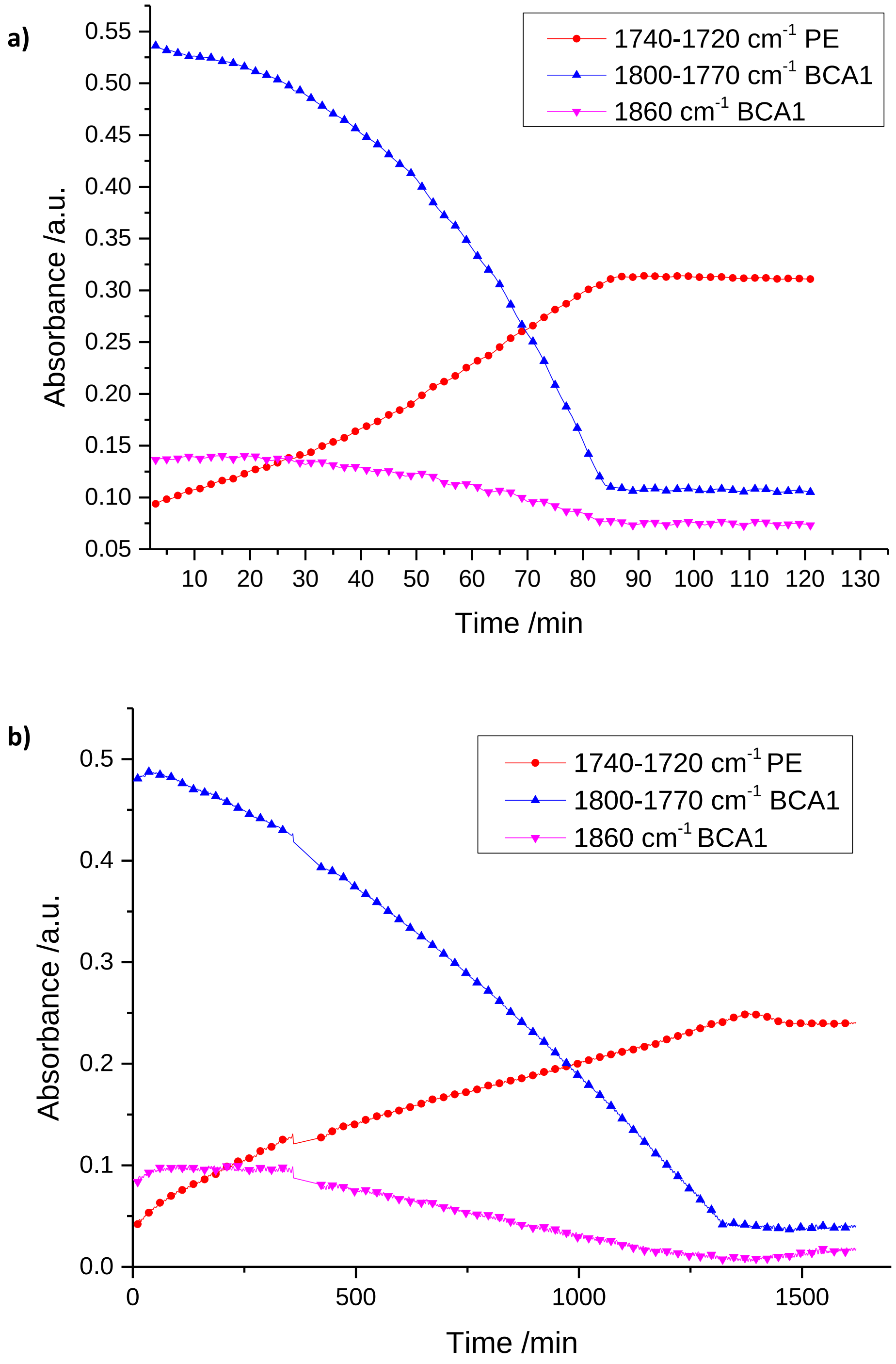

Figure S1: Illustrates changes in the intensity of certain IR frequency signals during the ROCOP of BCA1 and CHO using catalyst **1** (a) and catalyst **2** (b). Reaction conditions: catalyst:BCA1:CHO = 1:100:1000 at 100 °C under N_2. The data points missing at 300 mins in plot (b) is due to a power cut.

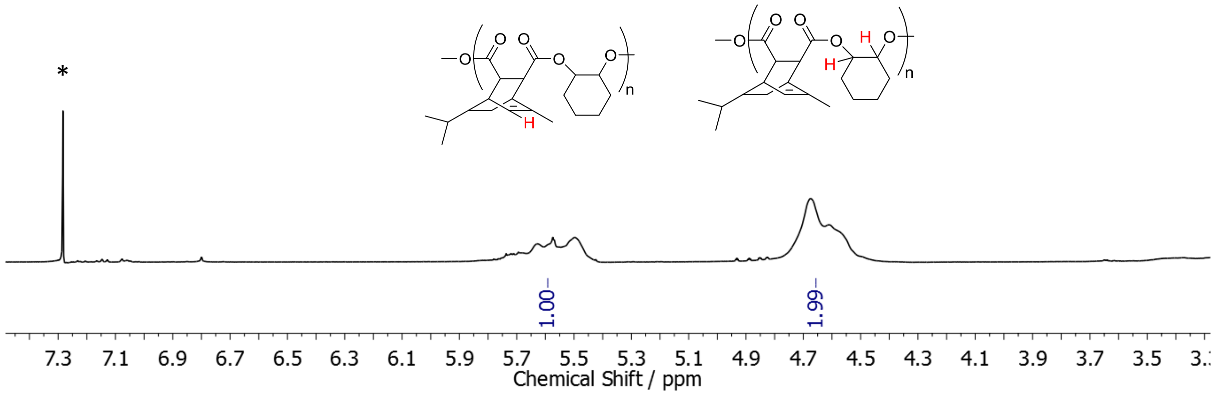

Figure S2: ^{1}H NMR spectra of copolymerization of BCA1/CHO with catalyst **1**. Reaction conditions: catalyst:BCA1:CHO = 1:100:1000 at 100 °C under N_2.

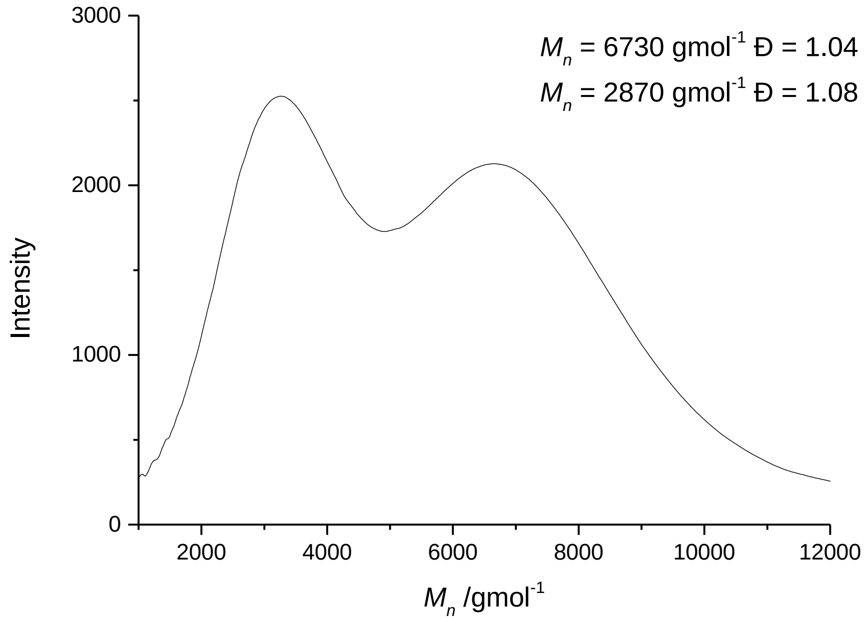

Figure S3: SEC trace of the PE produced during the copolymerization reaction between catalyst **1**/BCA1/CHO at 1:100:1000 at 100 °C.

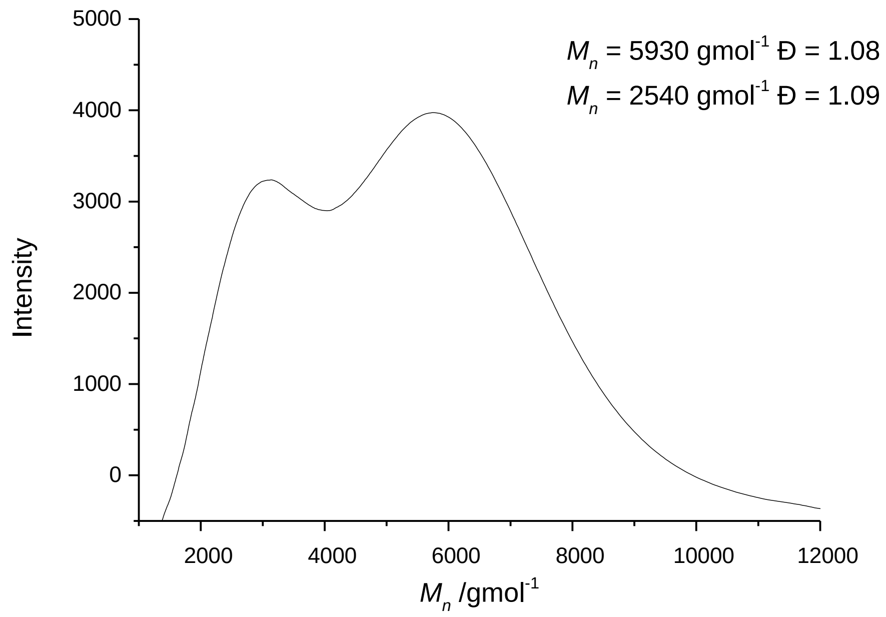

Figure S4: SEC trace of the PE produced during the copolymerization reaction between catalyst **2**/BCA1/CHO at 1:100:1000 at 100 °C.

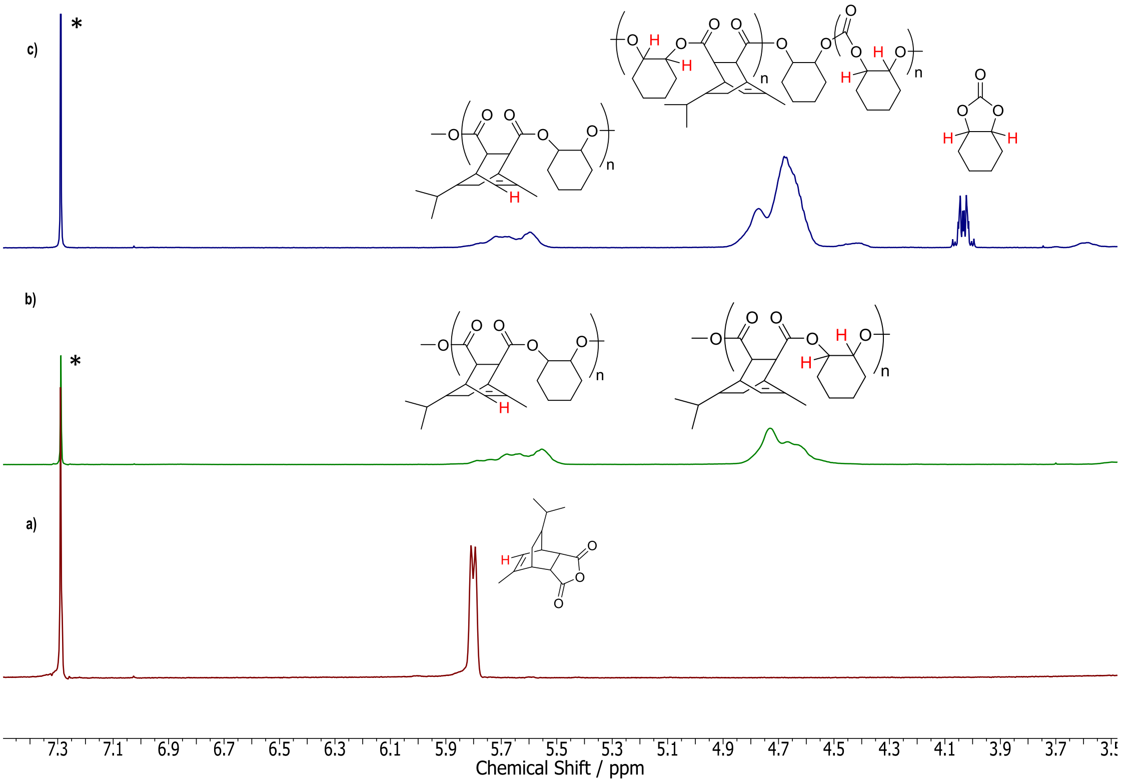

Figure S5: ^{1}H NMR spectra of aliquots taken during the terpolymerization of BCA1/CHO/CO_2 with catalyst **2**. Reaction conditions: catalyst:BCA1:CHO = 1:100:1000 at 100 °C under 1 bar pressure of CO_2. (a) ^{1}H NMR spectrum of BCA1. (b) ^{1}H NMR spectrum of the first aliquot after the formation of the polyester block (PE). (c) ^{1}H NMR spectrum of second aliquot taken after the formation of the polycarbonate block (polycyclohexene carbonate). All spectra were recorded in $CDCl_3$.

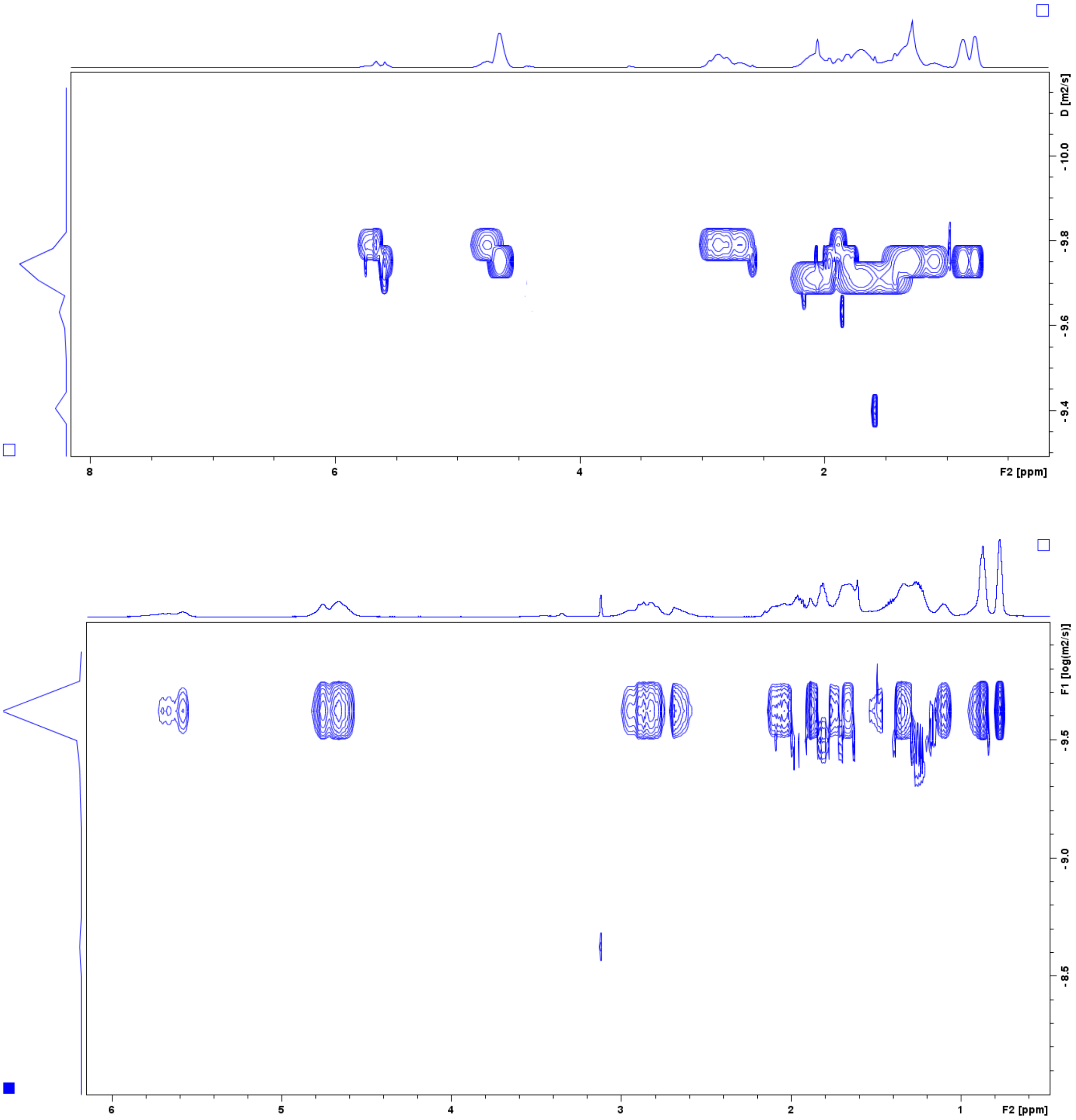

Figure S6: DOSY spectrum of polyester-polycarbonate block copolymer produced by catalyst **2** in $CDCl_3$ (top) and of a polyester/polycarbonate mixture recorded in $CDCl_3$ (bottom).

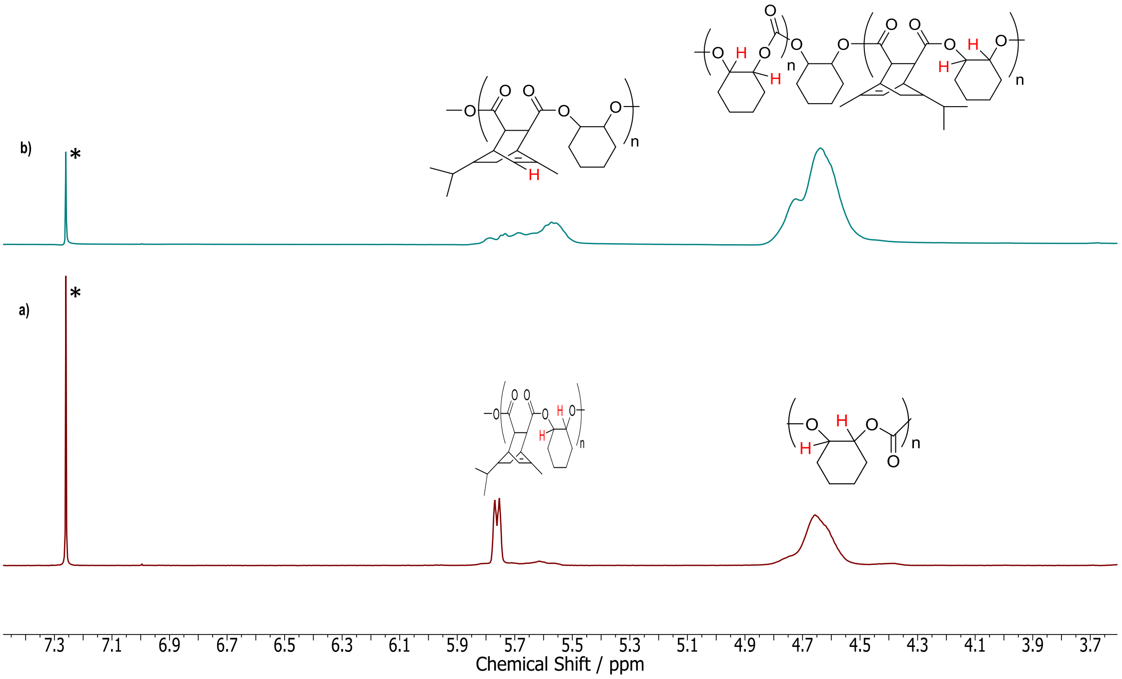

Figure S7: ^{1}H NMR spectra of aliquots taken during the polymerization of BCA1/CHO/CO_2 with catalyst **1**. Reaction conditions: catalyst:BCA1:CHO = 1:100:1000 at 100 °C under 1 bar pressure of CO_2. (a) ^{1}H NMR spectrum of the first aliquot after the formation of the polycarbonate block (PCHC). (b) ^{1}H NMR spectrum of second aliquot taken after the formation of the polyester block (PE), which was promoted by degassing the reaction vessel and replacing CO_2 with N_2. All spectra were recorded in $CDCl_3$.

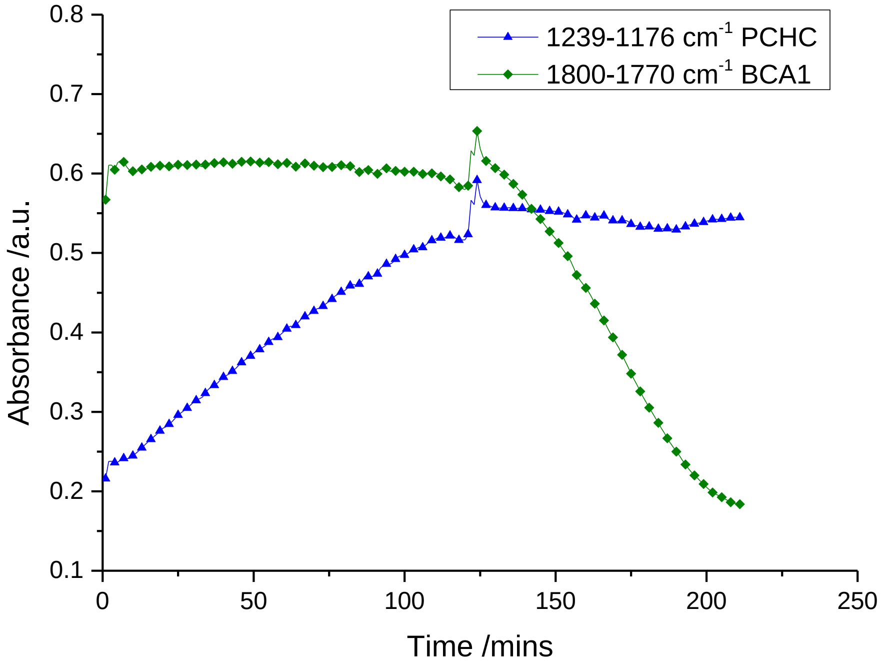

Figure S8: Illustrates changes in the intensity of certain IR frequency signals during the polymerization of BCA1, CHO, CO_2 using catalyst **1**. Reaction conditions: catalyst **1**:BCA1:CHO = 1:200:800 under 1 bar CO_2 pressure at 100 °C. The reaction mixture was degassed and the gas feed was changed from CO_2 to N_2 at 125 minutes. Where PCHC = polycyclohexene carbonate (polycarbonate).

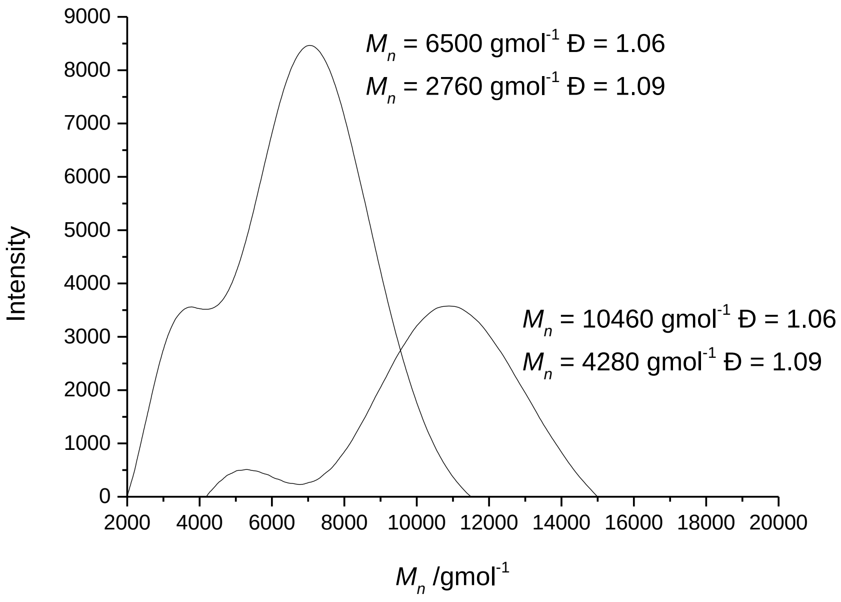

Figure S9: SEC trace of the aliquots taken during the terpolymerization reaction between catalyst **1**/BCA1/CHO/CO_2 at 1:100:1000:1 atm at 100 °C.

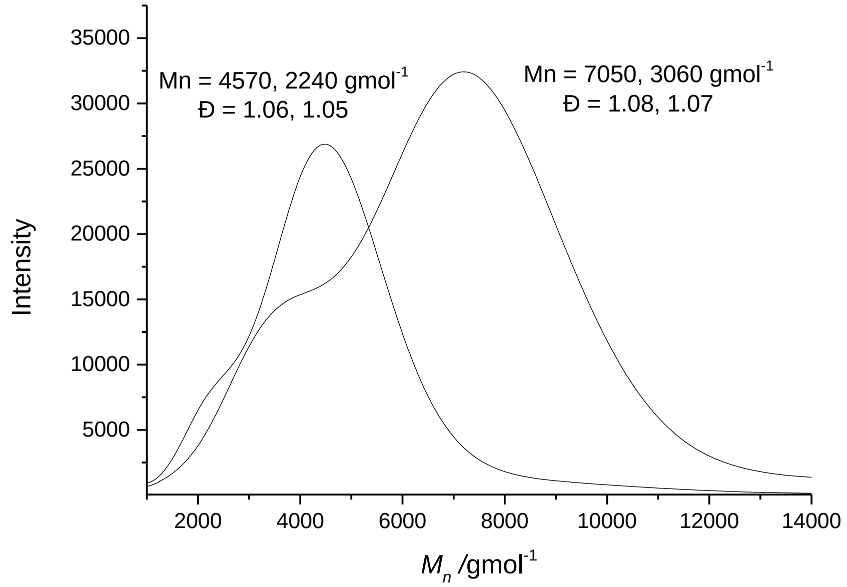

Figure S10: SEC trace of the aliquots taken during the terpolymerization reaction between catalyst **2**/BCA1/CHO/CO_2 at 1:100:1000:1 atm at 100 °C.

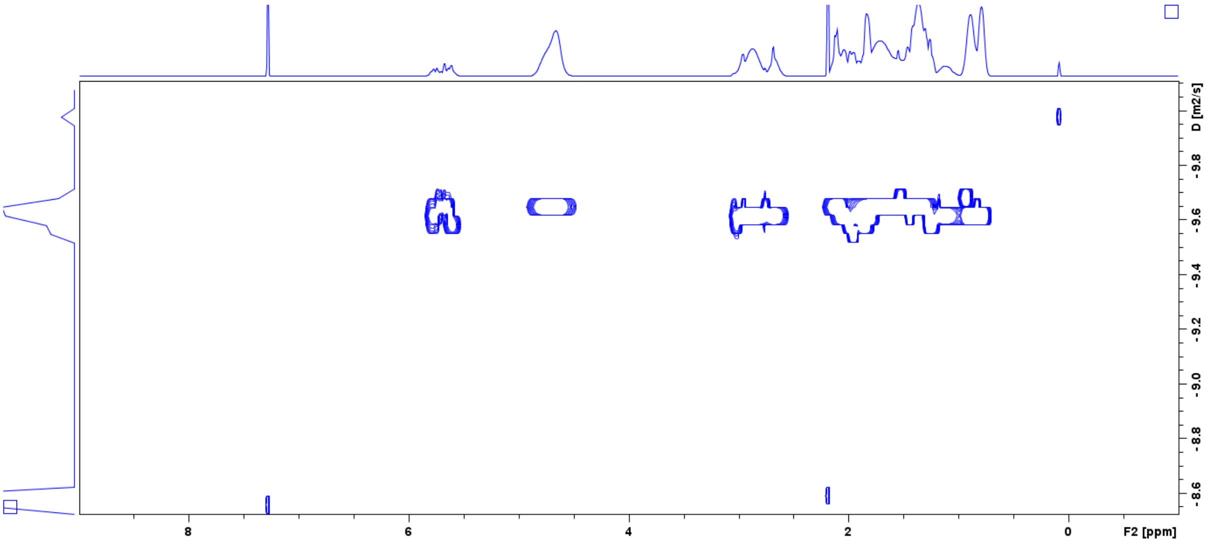

Figure S11: DOSY spectrum of polycarbonate-polyester block copolymer produced by catalyst **1** in $CDCl_3$.

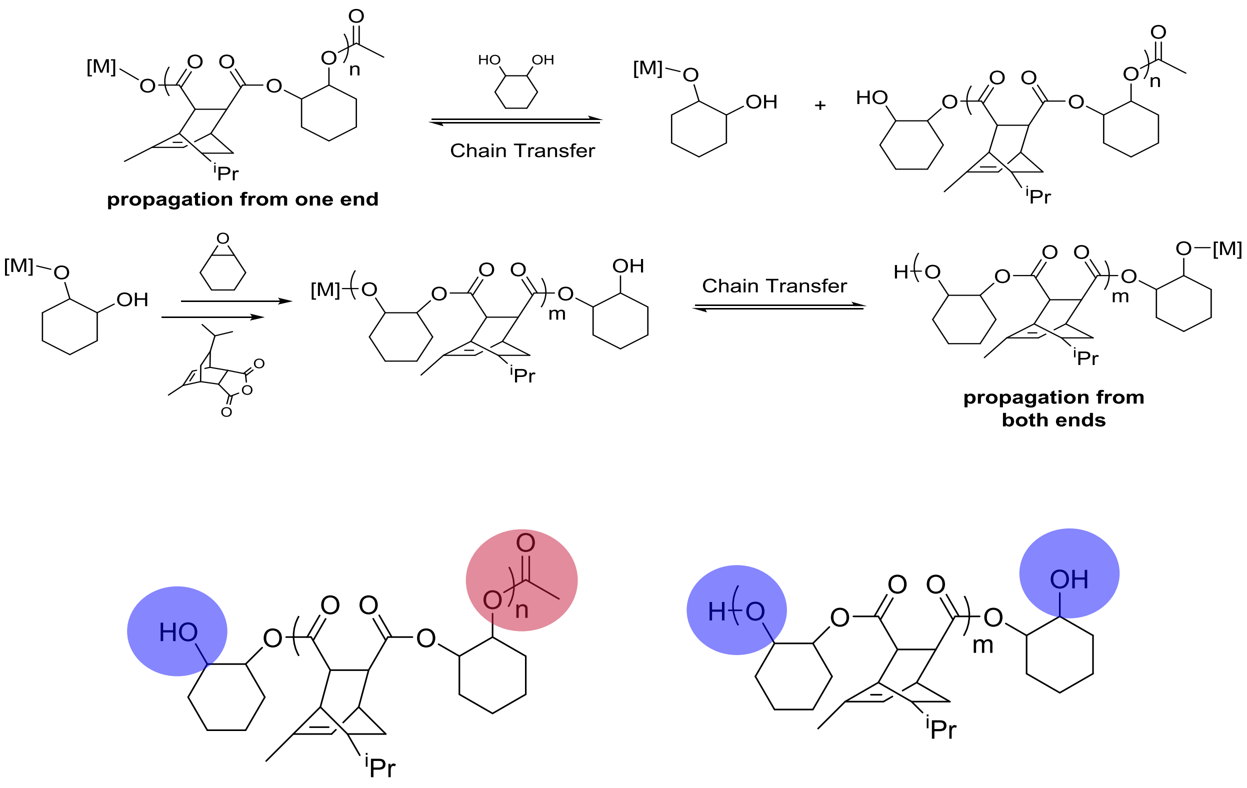

Figure S12: Illustrates the chain transfer reactions that occur during the copolymerization reactions with cyclohexane-1.2-diol (CHD) as the chain transfer agent (CTA). Two possible polymer series can form either α-acetyl-ω-hydroxyl end-capped (LHS) or α,ω-di-hydroxyl end-capped (RHS).

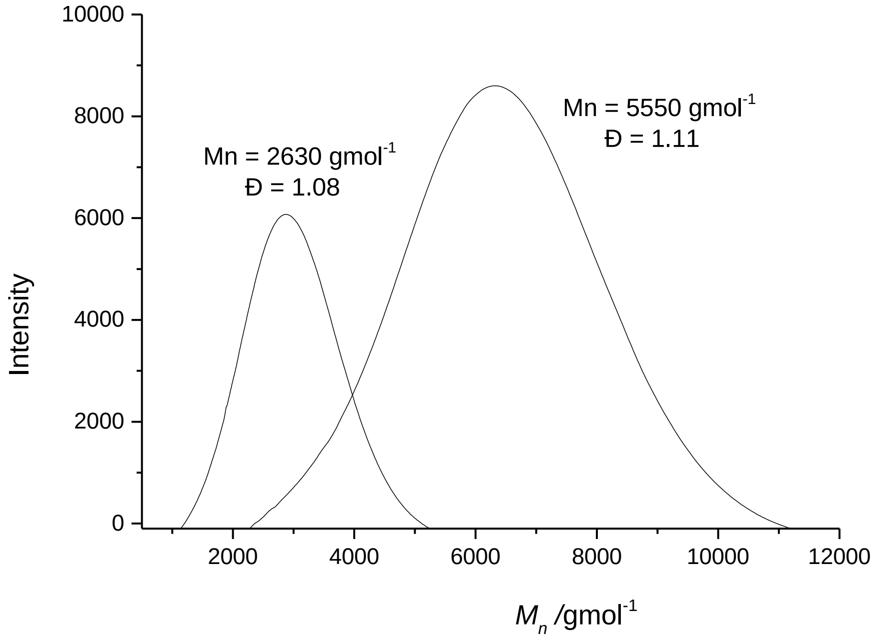

Figure S13: SEC trace of the aliquots taken during the 'switch' chemistry reaction between BCA1/CHO/CO_2/N_2 using catalyst **1** and 20 equivalents of cyclohexane-1.2-diol (CHD) at 100 °C and 1 bar CO_2 pressure.

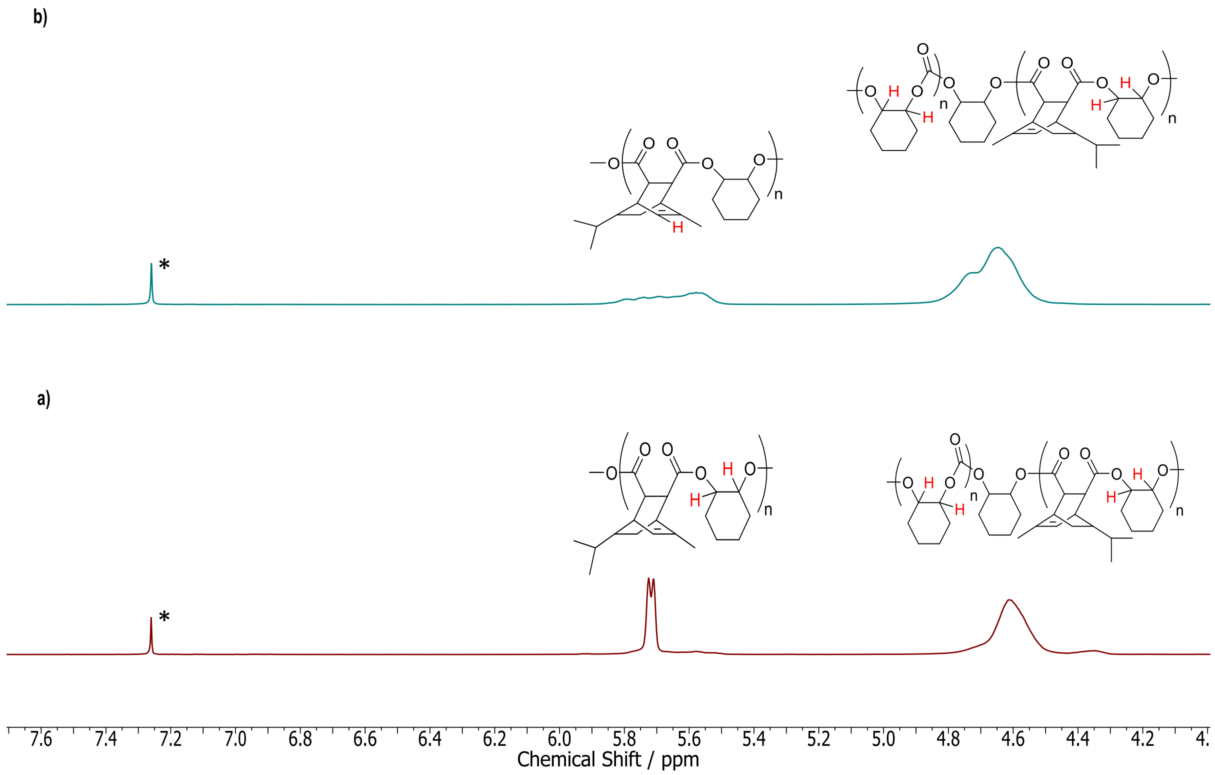

Figure S14: ^{1}H NMR spectra of aliquots taken during the reaction between BCA1/CHO/CO_2/N_2 using catalyst **1** and 20 equivalents of cyclohexane-1.2-diol (CHD) at 100 °C and 1 bar CO_2 pressure. (a) ^{1}H NMR spectrum of the first aliquot after the formation of the polycarbonate block (PCHC). (b) ^{1}H NMR spectrum of second aliquot taken after the formation of the polyester block (PE), which was promoted by degassing the reaction vessel and replacing CO_2 with N_2. All spectra were recorded in $CDCl_3$.

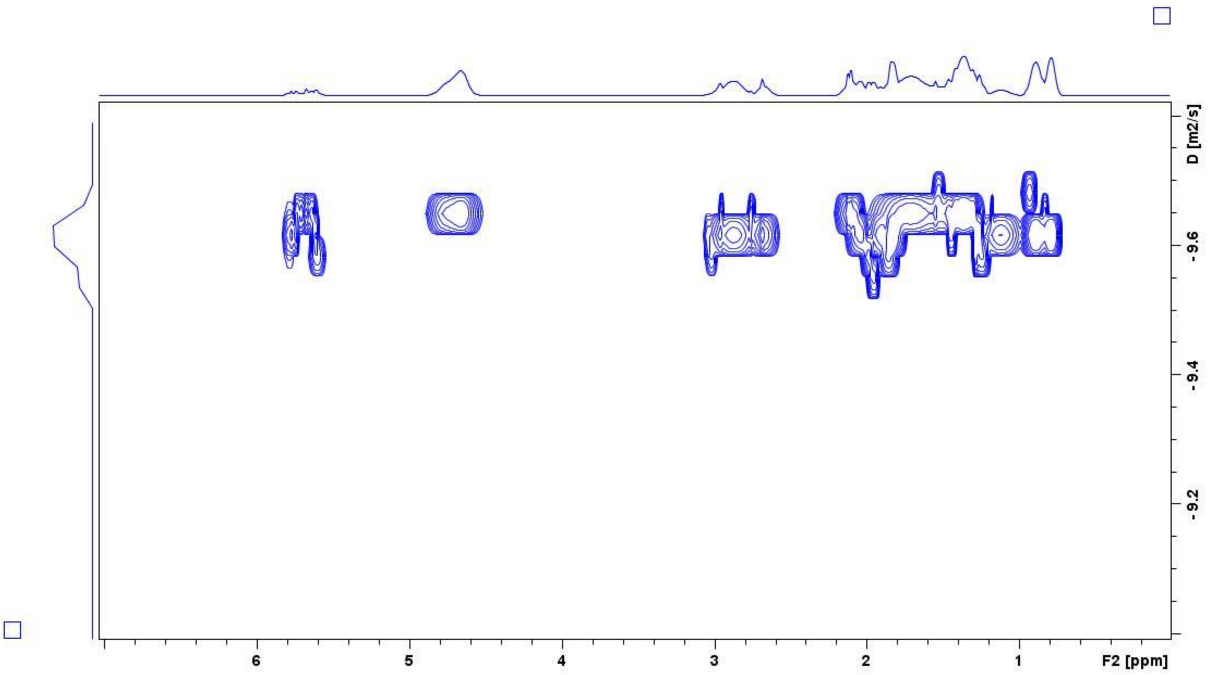

Figure S15: DOSY spectrum of polycarbonate-polyester block copolymer produced by catalyst **1** with 20 equivalents of cyclohexane-1.2-diol (CHD) in $CDCl_3$.

a)

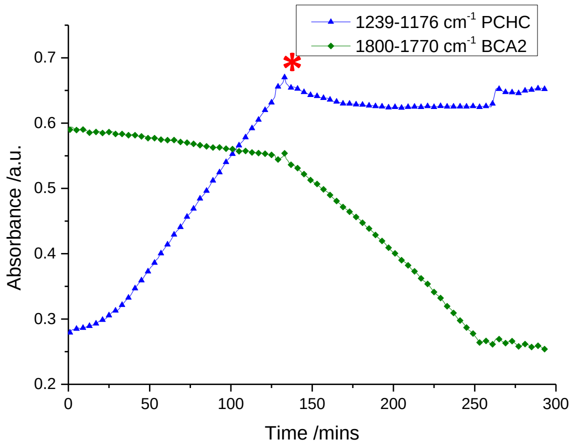

b)

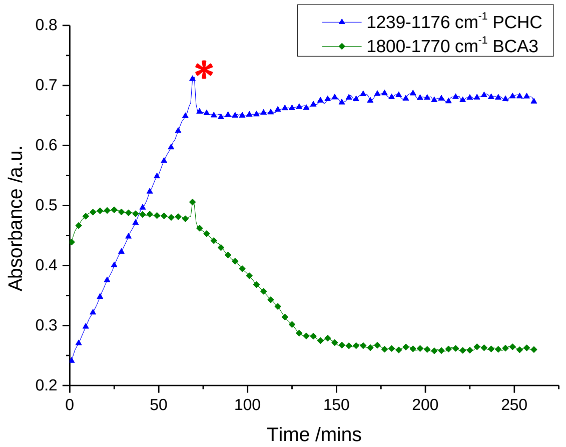

c)

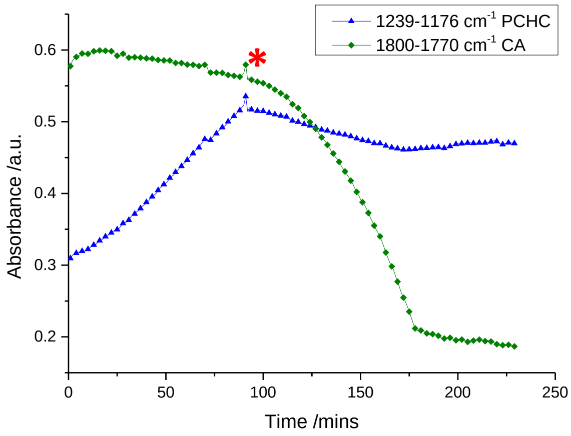

Figure S16: Illustrates changes in the intensity of certain IR frequency signals during the reactions between less sterically hindered bicyclic anhydrides (BCA2, BCA3 and CA), CHO, CO_2 and N_2. Reaction conditions: catalyst **1**:anhydride:CHO = 1:100:1000 under 1 bar CO_2 pressure at 100 °C. The reaction mixture was degassed and the gas feed was changed from CO_2 to N_2 at the point marked with an asterix. Where PCHC = polycyclohexene carbonate (polycarbonate).

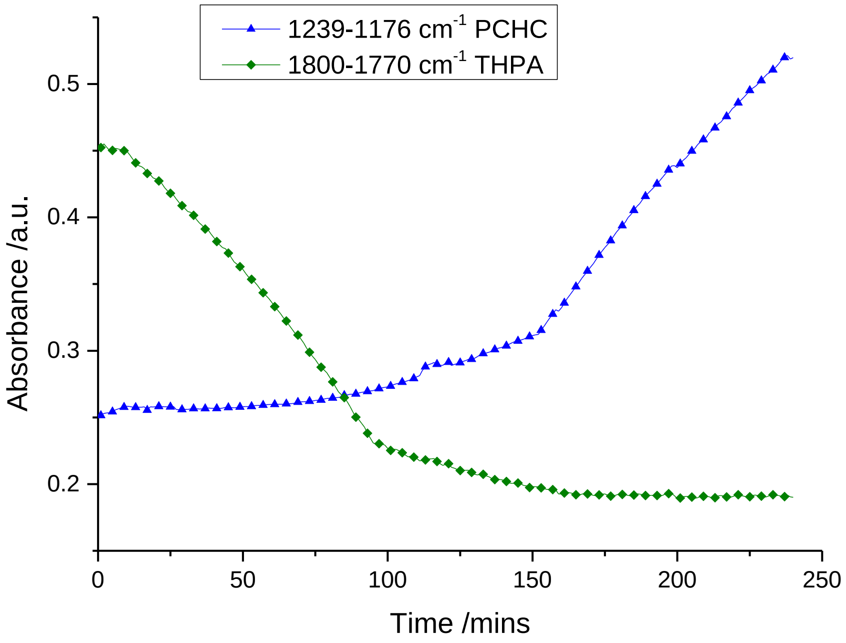

Figure S17: Illustrates changes in the intensity of certain IR frequency signals during the terpolymerization of THPA, CHO and CO_2. Reaction conditions: catalyst **1**:THPA:CHO = 1:100:1000 under 1 bar CO_2 pressure at 100 °C. Where PCHC = polycyclohexene carbonate (polycarbonate). The slight increase in intensity of the signal at 1239-1176 cm^{-1} between 80-150 mins is due to the concentration of anhydride being too low and thus CO_2 insertion becomes competitive with anhydride insertion and thus a gradient block forms between the PCHC-PE block copolymer.

Table S1: Polymerizations of anhydride (BCA1), epoxide (CHO) and CO2 catalysed by **1** and **2**.

**Entry**	**Cat.**	**t (h)**	**% ester linkages (% carbonate links in the block copolymers)**	**MW (Đ)**	**MW (Đ)from fitting**	**R^2**
1	1	1.3	> 99	5930 (1.08)<br>2530 (1.09)	7750 (1.14)<br>4180 (1.42)	0.99801
2	2	22	> 99	6730 (1.04)<br>2870 (1.08)	7460 (1.08)<br>4320 (1.24)	0.99948
3a	1	1.16	0 (100 % PC)	6500 (1.06)<br>2760 (1.09)	7810 (1.15)<br>4410 (1.46)	0.99928
3b	1	3.16	34 (66 % PC)	10,460 (1.08)<br>4280 (1.06)	11,800 (1.13)<br>6440 (1.34)	0.99878
4a	2	22	100 (0 % PC)	2240 (1.05)<br>4570 (1.06)	3040 (1.23)<br>4860 (1.07)	0.99974
4b	2	27.8	35 (65 % PC)	3060 (1.07)<br>7050 (1.08)	4950 (1.31)<br>7830 (1.10)	0.99961
5a	1	6	0 (100 % PC)	2630 (1.08)	3020 (1.09)	0.98471
5b	1	22.8	49 (51 % PC)	5550 (1.11)	6340 (1.11)	0.97916

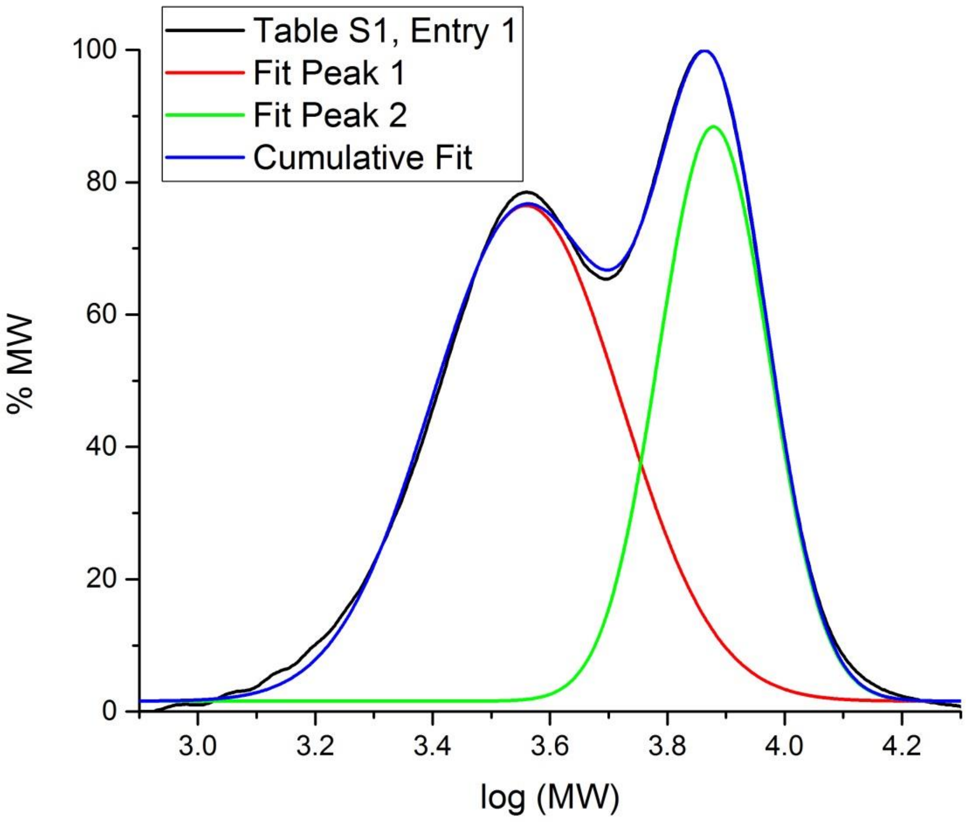

Figure S18: SEC trace (black) and SEC trace fitting (red, green and blue) of the PE produced during the copolymerization reaction between catalyst **1**/BCA1/CHO at 1:100:1000 at 100 °C (t = 1.3 h).

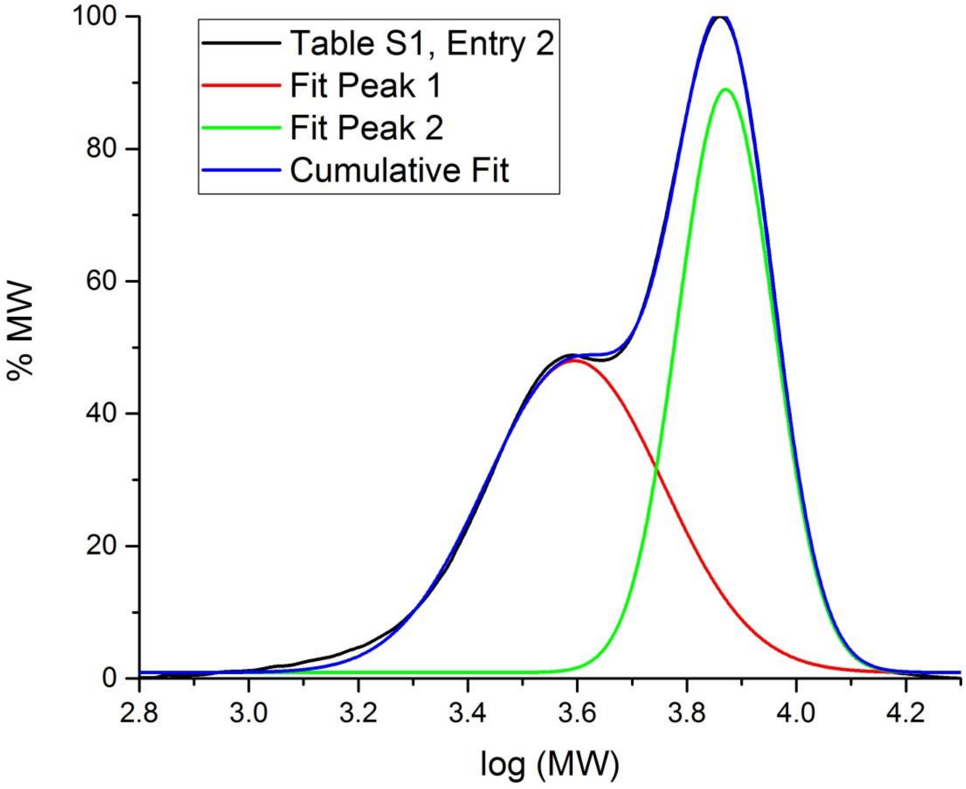

Figure S19: SEC trace (black) and SEC trace fitting (red, green and blue) of the PE produced during the copolymerization reaction between catalyst **2**/BCA1/CHO at 1:100:1000 at 100 °C (t = 22 h).

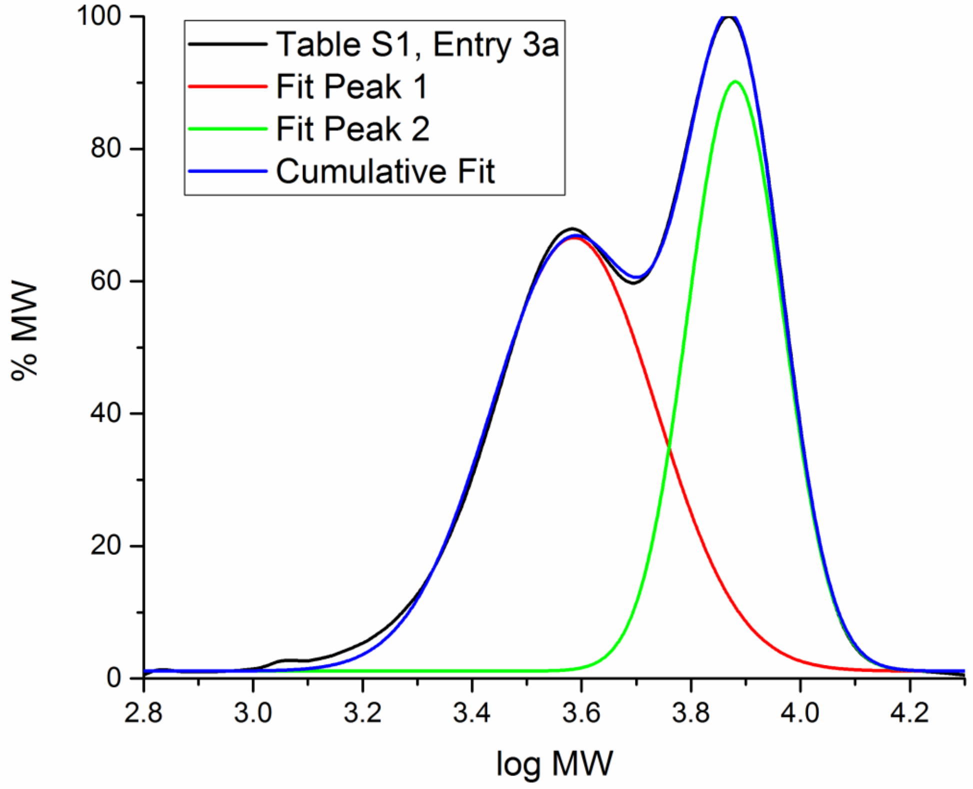

Figure S20: SEC trace (black) and SEC trace fitting (red, green and blue) of the PC produced during the copolymerization reaction between catalyst **1**/BCA1/CHO at 1:100:1000 at 100 °C (t = 1.16 h).

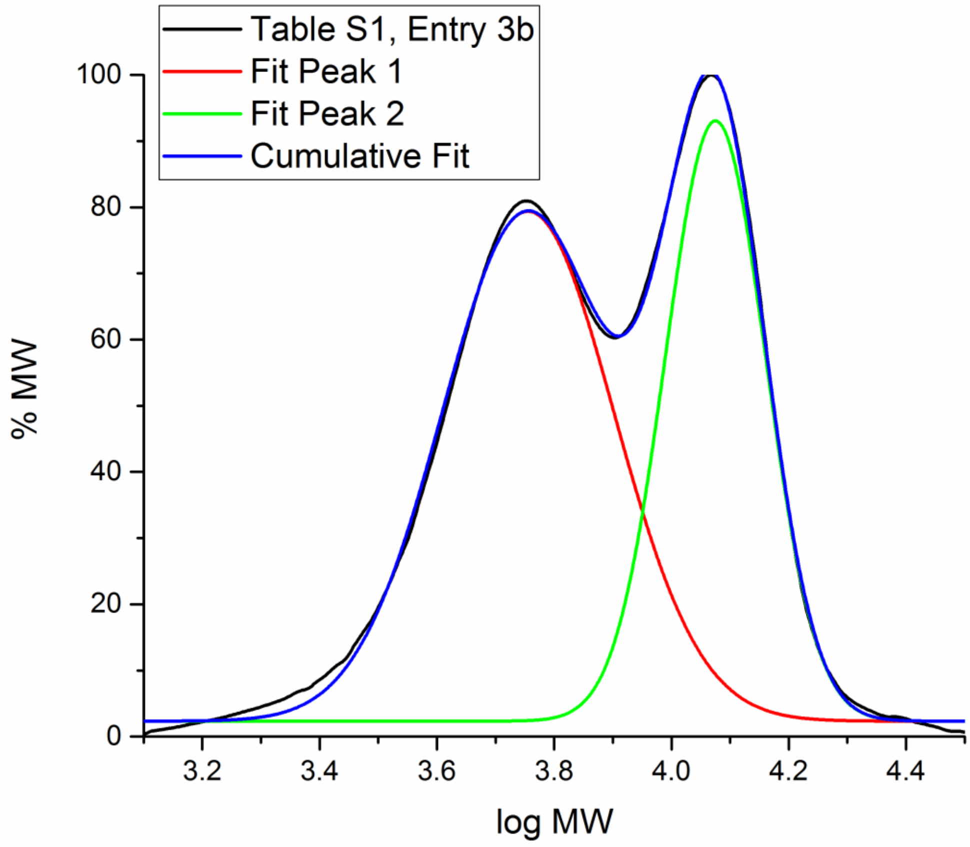

Figure S21: SEC trace (black) and SEC trace fitting (red, green and blue) of the block co-polymer produced during the copolymerization reaction between catalyst **1**/BCA1/CHO at 1:100:1000 at 100 °C (t = 3.16 h).

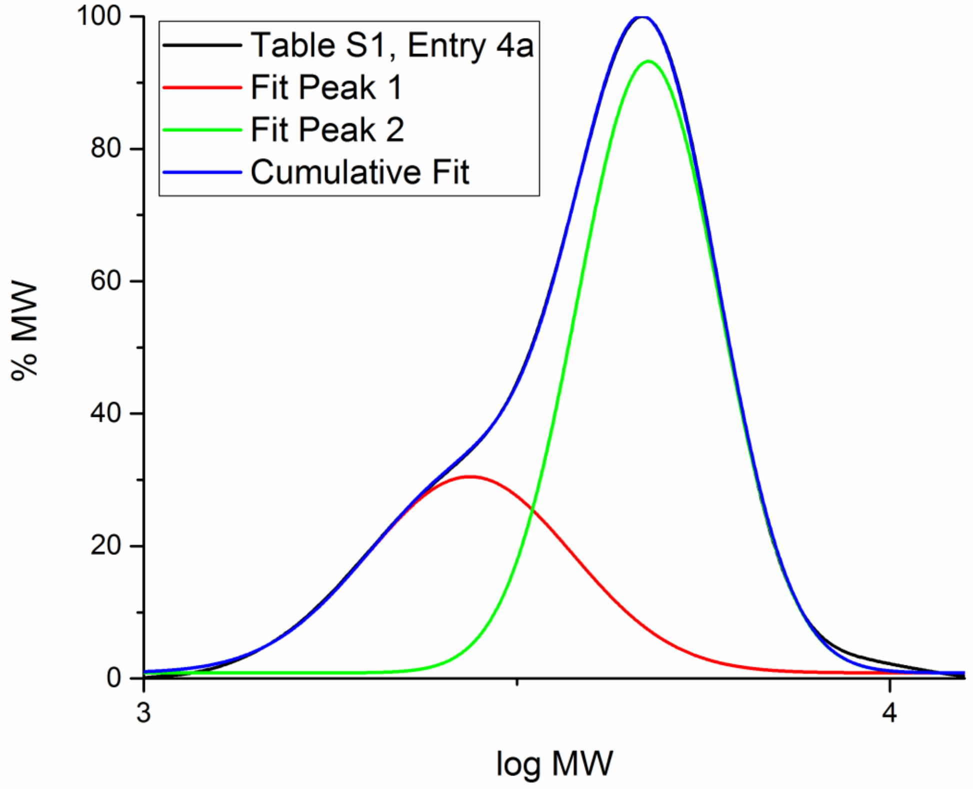

Figure S22: SEC trace (black) and SEC trace fitting (red, green and blue) of the PE produced during the copolymerization reaction between catalyst **2**/BCA1/CHO at 1:100:1000 at 100 °C (t = 22 h).

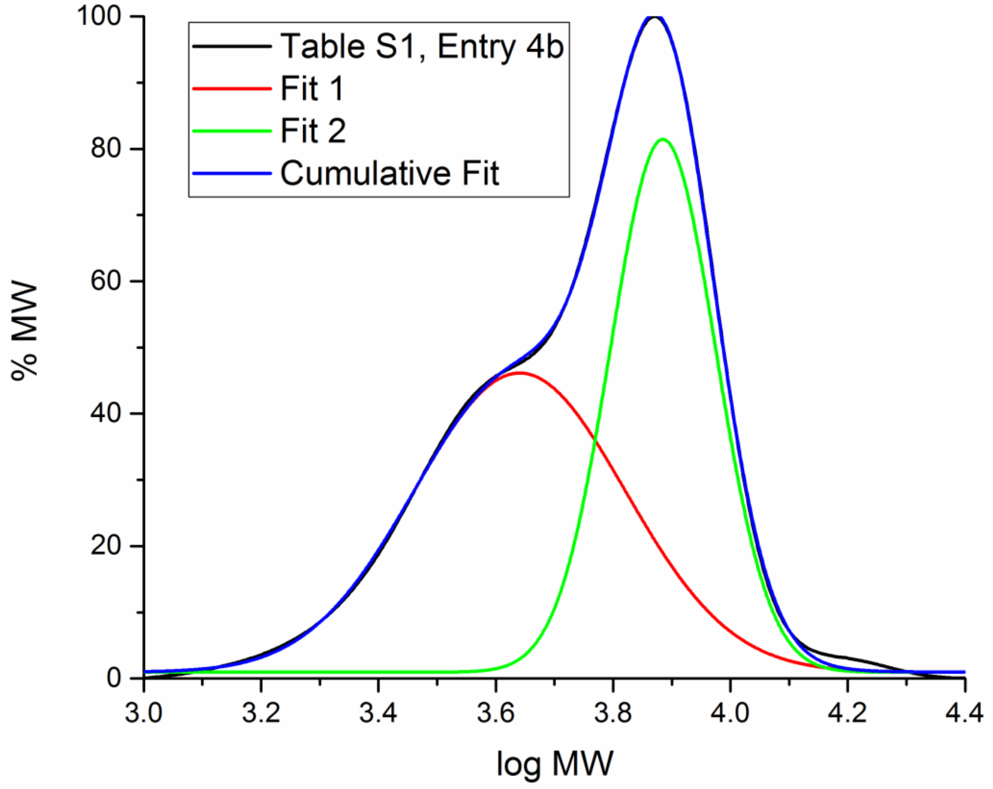

Figure S23: SEC trace (black) and SEC trace fitting (red, green and blue) of the block copolymer produced during the copolymerization reaction between catalyst **2**/BCA1/CHO at 1:100:1000 at 100 °C (t = 27.8 h).

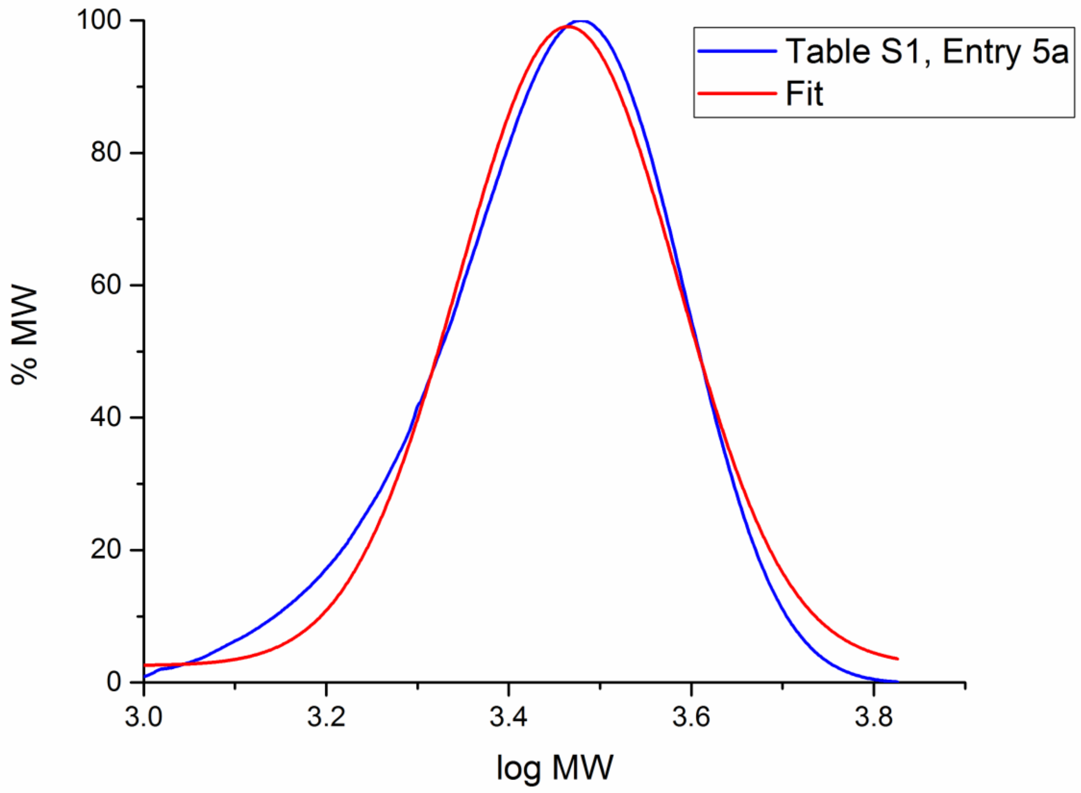

Figure S24: SEC trace (blue) and SEC trace fitting (red) of the PE produced during the copolymerization reaction between catalyst **1**/CHD/BCA1/CHO at 1:20:400:1500 and $p^0(CO_2)$=1 atm at 100 °C (t = 6 h).

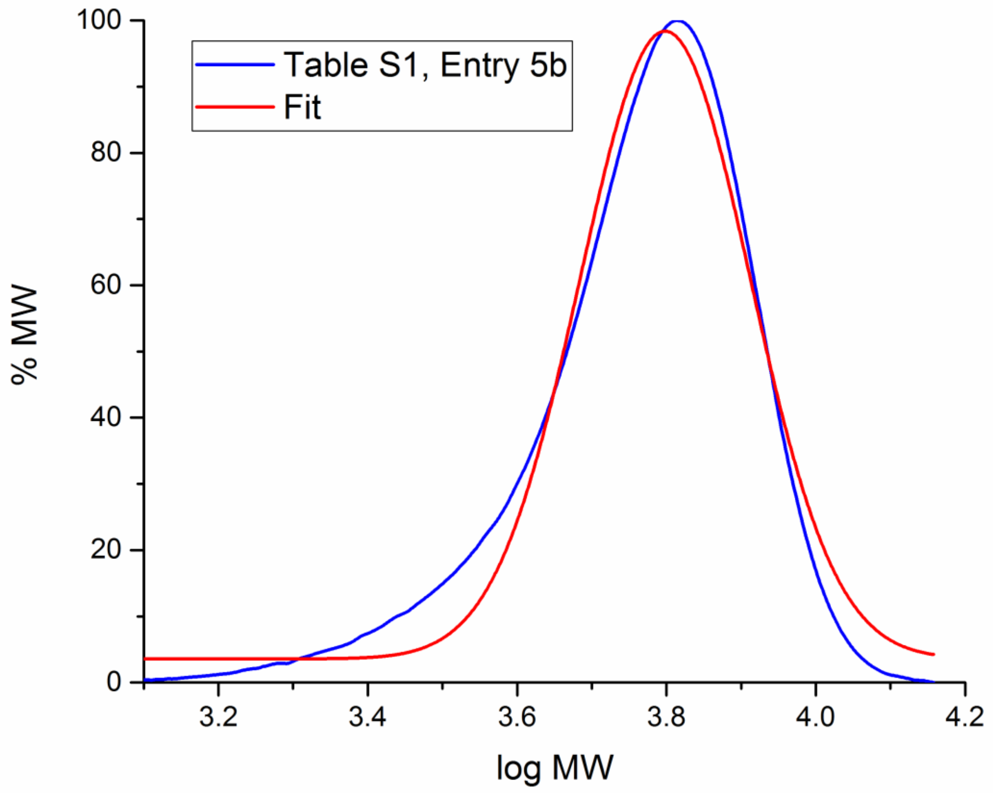

Figure S25: SEC trace (blue) and SEC trace fitting (red) of the block copolymer produced during the copolymerization reaction between catalyst **1**/CHD/BCA1/CHO at 1:20:400:1500 and $p^0(CO_2)$=1 atm at 100 °C (t = 22.8 h).

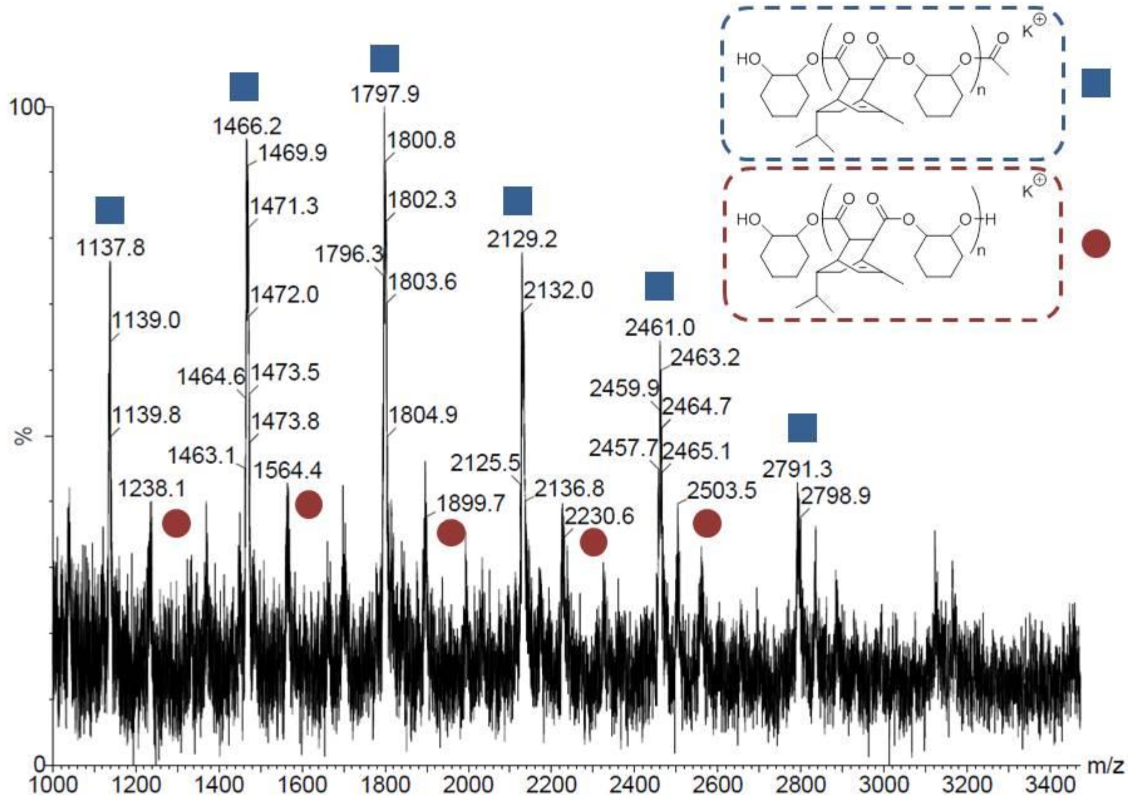

Figure S26: The MALDI-ToF spectrum of the polyester formed by complex **1** (Table 1 and S1, Entry 2).

References

1.(a) Kember, M. R.; Knight, P. D.; Reung, P. T. R.; Williams, C. K., *Angew. Chem. Int. Ed.* **2009**, 931-933; (b) Kember, M. R.; Williams, C. K., *J. Am. Chem. Soc.* **2012,** *134*, 15676-15679.
2.Dakshinamoorthy, D.; Weinstock, A. K.; Damodaran, K.; Iwig, D. F.; Mathers, R. T., *ChemSusChem* **2014,** *7*, 2923-2929.
